# Ultrafast Two‐Color X‐Ray Emission Spectroscopy Reveals Excited State Landscape in a Base Metal Dyad

**DOI:** 10.1002/advs.202404348

**Published:** 2024-08-05

**Authors:** Michal Nowakowski, Marina Huber‐Gedert, Hossam Elgabarty, Aleksandr Kalinko, Jacek Kubicki, Ahmet Kertmen, Natalia Lindner, Dmitry Khakhulin, Frederico A. Lima, Tae‐Kyu Choi, Mykola Biednov, Lennart Schmitz, Natalia Piergies, Peter Zalden, Katharina Kubicek, Angel Rodriguez‐Fernandez, Mohammad Alaraby Salem, Sophie E. Canton, Christian Bressler, Thomas D. Kühne, Wojciech Gawelda, Matthias Bauer

**Affiliations:** ^1^ Chemistry Department and Center for Sustainable Systems Design (CSSD) Faculty of Science Paderborn University Warburger Straße 100 33098 Paderborn Germany; ^2^ Deutsches Elektronen‐Synchrotron DESY 22607 Notkestr. 85 Hamburg Germany; ^3^ Faculty of Physics Adam Mickiewicz University, Poznań Uniwersytetu Poznańskiego 2 Poznań 61‐614 Poland; ^4^ European X‐Ray Free‐Electron Laser Facility GmbH 22869 Holzkoppel 4 Schenefeld Germany; ^5^ PAL‐XFEL Jigok‐ro 127–80 Pohang 37673 Republic of Korea; ^6^ Institute of Nuclear Physics Polish Academy of Sciences Kraków 31‐342 Poland; ^7^ The Hamburg Centre for Ultrafast Imaging 22761 Luruper Chaussee 149 Hamburg Germany; ^8^ Fachbereich Physik Universität Hamburg 22607 Notkestraße 9–11 Hamburg Germany; ^9^ Department of Chemistry Technical University of Denmark Kongens Lyngby DK‐2800 Denmark; ^10^ IMDEA Nanociencia Calle Faraday 9 Madrid 28049 Spain; ^11^ Departamento de Química Universidad Autónoma de Madrid Campus Cantoblanco Madrid 28047 Spain; ^12^ Center for Advanced Systems Understanding (CASUS) Helmholtz‐Zentrum Dresden‐Rossendorf 02826 Untermarkt 20 Görlitz Germany; ^13^ Institute of Artificial Intelligence, Chair of Computational System Sciences Technische Universität Dresden 01187 Helmholtzstr. 10 Dresden Germany

**Keywords:** charge transfer, electron dynamics, excited state, MLCT, XFEL, X‐ray emission

## Abstract

Effective photoinduced charge transfer makes molecular bimetallic assemblies attractive for applications as active light‐induced proton reduction systems. Developing competitive base metal dyads is mandatory for a more sustainable future. However, the electron transfer mechanisms from the photosensitizer to the proton reduction catalyst in base metal dyads remain so far unexplored. A Fe─Co dyad that exhibits photocatalytic H_2_ production activity is studied using femtosecond X‐ray emission spectroscopy, complemented by ultrafast optical spectroscopy and theoretical time‐dependent DFT calculations, to understand the electronic and structural dynamics after photoexcitation and during the subsequent charge transfer process from the Fe^II^ photosensitizer to the cobaloxime catalyst. This novel approach enables the simultaneous measurement of the transient X‐ray emission at the iron and cobalt K‐edges in a two‐color experiment. With this methodology, the excited state dynamics are correlated to the electron transfer processes, and evidence of the Fe→Co electron transfer as an initial step of proton reduction activity is unraveled.

## Graphical abstract

A prototypic photosensitizer‐catalyst system produces hydrogen more efficiently than the constituting catalyst. Upon visible light absorption, a complex electronic cascade that facilitates the reaction is activated and tracked by X‐ray emission spectroscopy. The charge transfer from the Fe center to the Co catalyst allows to maintain increased electron density required for the reaction on the Co catalyst to happen. The electronic cascade includes nuclear oscillations due to the excited states intersection.

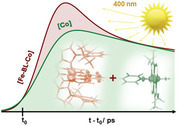

## 1 Introduction

Fe^II^ complexes can operate as light‐harvesting components in bimetallic molecular assemblies (dyads). They convert solar to chemical energy by ultrafast charge transfer (CT) to a second catalyst metal for photocatalytic proton reduction.^[^ ^1^ ^]^ In terms of sustainability, the second metal should at least be abundant. Cobaloxime fulfills this requirement.^[^ ^2^, ^3^, ^4^ ^]^ While recent reports highlighted some devastating human and environmental aspects of cobalt mining, this underscores the fact that sustainable solutions still rely on unsustainable practices, which must be concomitantly addressed in a wider scope. It is probably the case that the increased demand for alternatives likely sets into motion similar human/environment abuse mechanics, merely leading to “environmental problem‐shifting”^[^ ^5^ ^]^ which highlights the multi‐dimensionality of the sustainability problem. The quest for clean energy is by far not only a scientific one, as there are inevitable political, socioeconomic, and ethical aspects that are always entangled and must be tackled alongside the scientific efforts.

Despite the reported short lifetimes of metal‐to‐ligand charge transfer (MLCT) states in iron(II) photosensitizers, photocatalytic proton reduction activity was reported for Fe^II^─Co^III^ dyads.^[^ ^6^ ^]^ However, its activity remains mysterious, as no charge transfer from the Fe to the Co center could be observed experimentally. Thus, rational improvement of Fe─Co dyads requires a radically different approach to understand the working principle. A major challenge is the ultrafast photophysics at the Fe^II^ center,^[^ ^7^ ^]^ and the difficulty of monitoring CT from the photosensitizer to the catalyst with element specificity in real‐time.^[^ ^1^, ^8^, ^9^ ^]^ Upon photoexcitation, the excited state dynamics in dyads can involve MLCT and ligand‐to‐metal charge transfer states (LMCT), metal‐centered (MC), and ligand‐mediated metal‐to‐metal charge‐transfer states (M'MCT).^[^ ^10^, ^11^ ^]^ The de‐excitation cascade is an interplay between MC and CT states modulated by intramolecular vibrational energy dissipation, strong spin‐orbit coupling such as intersystem crossings (ISC), and internal conversions (IC).^[^ ^9^, ^12^ ^]^ The fundamental principles guiding the properties are typically identified using laser spectroscopy.^[^ ^13^, ^14^ ^]^ Noble metal complexes exhibit long‐lived CT states, which can easily be followed with optical spectroscopy due to the associated intense absorption bands in the UV–vis range.^[^ ^15^ ^]^ However, in most iron photosensitizers, the smaller ligand field splitting leads to an unfavoured energetic order of E(MLCT) > E(MC).^[^ ^2^ ^]^ In addition, MC states are hardly accessible in the UV–vis spectral range.^[^ ^16^ ^]^

In contrast, X‐ray emission spectroscopy (XES) is very sensitive to MC states due to the localized character of the core levels.^[^ ^17^ ^]^ Both Kα (2*p*→1*s*) and Kβ (3*p*→1*s*) emission lines provide characteristic multiplicity signatures of the involved transient MC states.^[^ ^18^, ^19^, ^20^ ^]^ For monomeric iron carbene photosensitizers, femtosecond XES could uniquely reveal details of the excited states.^[^ ^14^, ^18^, ^21^ ^]^ The excited states of [Fe(bmip)_2_]^2+^ [bmip = 2,6‐bis(3‐methyl‐imidazole‐1‐ylidine)‐pyridine] show a branching pattern. A long‐lived ^3^MLCT dominates one path, while the second includes a rapid hot MLCT* to ^3^MC transition connected to bond oscillations in the form of wavepacket dynamics.^[^ ^18^, ^22^, ^23^, ^24^ ^]^ Ultrafast X‐ray absorption near edge structure spectroscopy (XANES) on photoactive Fe─Co Prussian blue analogs revealed that a spin transition at the Co center preceded CT between the Fe and Co center.^[^ ^25^ ^]^ More recently, the photoinduced M'MCT transition in a bimetallic Fe‐Ru assembly was shown to have a critical impact on solvent organization processes.^[^ ^26^ ^]^ Solvent rearrangement occurred in response to the M'MCT followed by back electron transfer in 62 fs, creating an upper limit for the charge transfer timescale.

These pioneering studies on bimetallic model complexes pave the way for the first application of ultrafast X‐ray spectroscopy to understand the working principle of a novel class of base metal dyads active in hydrogen generation.^[^ ^27^ ^]^ We demonstrate the unique potential of two‐color X‐ray emission spectroscopy (2C‐XES) in photocatalysis research. It allows for simultaneous, ultrafast detection of the Fe and Co Kα XES in an [Fe─BL─Co] assembly of a heteroleptic Fe^II^ photosensitizer with two different biscarbene‐pyridine ligands (C^N^C) connected to a cobaloxime catalyst via a bridging ligand (BL).^[^ ^6^ ^]^ The dynamics of the excited state decay are monitored at the Fe and Co sites to follow the departure of the charge from the photosensitizer and its arrival at the catalyst in real‐time. This approach eliminates uncertainties related to the charge transfer event timescale and sheds light on a possible charge transfer in base metal dyads.

## 2 Results and Discussion

**Figure 1:**
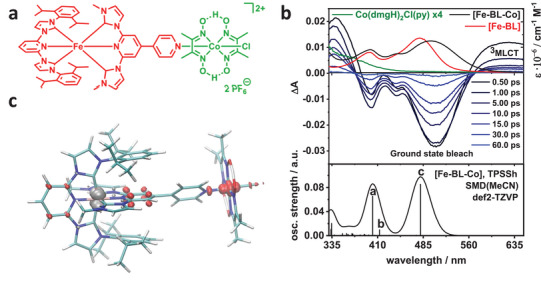
a) Structure of the [Fe─BL─Co] dyad; b) Top: Steady state UV–vis spectra for [Fe─BL] (red), [Co] (green) and [Fe─BL─Co] (black) overlapped with transient absorption spectra for selected delay times specified in the legend. The steady state UV–vis spectrum for [Co] was scaled by a factor of four to underline very weak ε values in the LMCT region. Bottom: TD‐DFT UV–vis spectrum for the dyad. c) Fe → Co CT (M'MCT): grey color indicates holes, red– electrons.

The dyad is synthesized by combining a heteroleptic tetra‐NHC Fe^II^ photosensitizer [Fe‐BL] coordinated by a 2,6‐bis[3‐(2,6‐diisopropylphenyl)imidazol‐2‐ylidene]pyridine and a 2,6‐bis(3‐methyl‐imidazol‐2‐ylidene)−4,4′‐bipyridine ligand (BL) with a Co^III^ cobaloxime catalyst, as presented in **Figure** **1**a.^[^ ^6^ ^]^ A 4,4′‐bipyridine (bpy) linker connects both metals with a distance of 11 Å.^[^ ^28^, ^29^ ^]^ The ground state optical absorption spectra of the dyad (black line) in comparison to the constituting components [Fe─BL] (red line) and cobaloxime (Co(dmgH)_2_Cl(py) ═ [Co], green line) are shown in Figure 1b. The top panel shows two absorption bands for the photosensitizer [Fe─BL] at 398 and 481 nm (Figure 1c, top). By the coordination of the cobaloxime in [Fe─BL─Co] (black line), the steady state UV–vis absorption spectrum of the dyad changes in a distinct manner: The 398 nm band remains unchanged while the 481 nm band is shifted to 497 nm, and a new band appears at 444 nm (Figure 1b, top). Cobaloxime itself shows only a weak absorption ≈400 nm. Measurements performed in solvents with different polarities indicate a small shift at the 400 nm peak, indicating a minor charge transfer nature of the 400 nm band (Figure S2.1, Supporting Information).

### 2.1 Quantum Chemical Assignment of the Optical Absorption Bands

In order to shed further light on the properties of the electronic excited states of [Fe─BL─Co], we resort to quantum chemical calculations. Here, one would preferably rely on the complete‐active‐space self‐consistent field (CASSCF)‐based methods, which provide a qualitatively correct description of excited states.^[^ ^30^ ^]^ The quantitative accuracy of CASSCF can be substantially improved by coupling with perturbative corrections, e.g., NEVPT2. The computational cost of these methods steeply increases with the number of correlated orbitals.^[^ ^31^ ^]^ In our case, we have found that the dyad molecule is too large for the method (Section S1, Supporting Information). The active space required to accurately compute the electronic spectrum of the photosensitizer included 14 electrons in 13 active orbitals, CASSCF(14,13). Performing NEVPT2 calculations including all the 12 *d*‐electrons together with the interacting ligand electrons at both transition metal centers would be computationally unfeasible.

Another highly popular alternative for the study of excited states is time‐dependent density functional theory (TDDFT). TDDFT has been successfully used to study *d* ^6^ transition metal complexes.^[^ ^32^ ^]^ This method however suffers from several disadvantages, especially with systems having charge transfer states, and one should not blindly trust TDDFT results without scrutiny.^[^ ^33^, ^34^ ^]^

To this end, we have benchmarked TDDFT UV–vis electronic spectra, using both the hybrid‐GGA B3LYP functional and the hybrid‐meta‐GGA TPSSh functional, against CASSCF‐NEVPT2 calculations of the photosensitizer (Section S1a, Supporting Information).

Our benchmark shows that TPSSh (a meta‐hybrid functional with 10% Hartree–Fock exchange) performs quantitatively better than B3LYP (a hybrid functional with 20% HF exchange). Rather than the exact positions of the peaks, more important to our benchmark is the nature of the underlying states and transitions. Here, we find very consistent behavior between both TDDFT functionals and CASSCF (Section S1a, Supporting Information for a detailed comparison).^[^ ^31^ ^]^Both techniques reveal that the bands in the experimentally measured spectrum at 398 and 481 nm in [Fe─BL] (Figure 1c, top, red) are a mixture of MLCT transitions from Fe^II^ to both the terminal and the BL.

Based on the benchmark, we rely on TPSSh/TDDFT for the computation of the UV–vis spectrum of the dyad [Fe─BL─Co] (Section S1c,f, Supporting Information), which is shown in the lower panel of Figure 1c. The experimentally observed 398 nm absorption band is described by transitions a and b (Section S1c, Supporting Information). Like in the photosensitizer, they are composed of MLCT transitions from iron to the terminal and bridging ligand. Additionally, the electron density is transferred from the Fe^II^ to the Co^III^ center along the bridging ligand in the form of an M'MCT transition (Section S1c, Supporting Information). The donor–acceptor contributions to the latter are shown in Figure 1c. The absorption at 497 nm is dominated by an MLCT transition to the bridging ligand together with a weak M'MCT contribution (transition c, Section S1c, Supporting Information). The shift of the 497 nm band in the dyad absorption spectrum compared to the photosensitizer absorption spectrum (481 nm) is well reproduced by TDDFT (480.6 vs 446.0 nm, Section S1b,c, Supporting Information). This is due to an increased charge transfer to the terminal pyridine ring (transition c in Figure 1c; Figure S1.4, Supporting Information) and revealed by charge transfer components obtained for the dyad (Section S1f, Supporting Information). Unfortunately, TDDFT could not resolve the 444 nm band in the dyad spectrum, giving only a low intensity transition that is not discernible under the envelope of the two major peaks. After re‐evaluating the former interpretation,^[^ ^6^ ^]^ it is apparent that this band is also present in the photosensitizer spectrum but overlaps with the 481 nm band.

Analysis of the DFT vertical excitations (Section S1b–e, Supporting Information) reveals that introducing the [Co] moiety causes significant directionality in the CT transitions. For [Fe─BL] ≈400 nm, the main MLCT transition is evenly distributed around the Fe, with only a 10% MLCT contribution directed toward the bridging ligand. On the other hand, for the same band in [Fe─BL─Co], we have from the [Fe] side two MLCT/M'MCT transitions, contributing 50% to the charge transfer excitation and being directed toward [BL] and [Co]. In addition to this CT in the direction [Fe]→[Co], the 330 nm peak of [Fe─BL─Co] shows an enhanced intensity compared to [Fe─BL]. This is the outcome of a superposition of the spectra of [Fe─BL] and [Co] but is also due to an MLCT/M'MCT transition, this time in the direction [Co]→[Fe], with the electron density equally shifting to [BL] and [Fe] by 40% each. These findings clearly show that, according to TDDFT, the two metal centers of the dyad communicate via the bridging ligand. Further experimental evidence is discussed below. While the relative contribution of M'MCT to the total transition moment is only minor in this case, the fact that the dyad exhibits such enhanced directionality is indeed very promising and suggests that structural modifications can enhance the M'MCT contribution.

### 2.2 Transient Absorption Spectroscopy

The experimentally measured transient absorption spectroscopy (TA) results for [Fe─BL─Co] are presented in Figure 1b. The ground state bleach occurs at 370–560 nm, and an excited‐state absorption is observed <370 and >560 nm. The transient absorption >560 nm is assigned to a ^3^MLCT state,^[^ ^6^ ^]^ and its kinetics are composed of three time constants (Figure S2.4 and Section S2, Supporting Information). The assignment of this particular band to ^3^MLCT state is based on the fact that the shape of the transient spectrum for energies lower than 560 nm is very similar to the differential UV–vis spectrum calculated for the ground state and oxidised compounds of similar structure. This is a strong indication of the dominating MLCT character.^[^ ^35^ ^]^ The first component (<100 fs) takes all coherent artifacts and possible ^1^MLCT contributions into account.^[^ ^36^ ^]^ The second component (*τ_2_* = 350 fs) can be ascribed to either the relaxation from the hot ^3^MLCT* to thermally relaxed ^3^MLCT^[^ ^37^, ^38^ ^]^ or to a ^1^MLCT → ^3^MLCT transition.^[^ ^12^, ^16^, ^18^, ^21^ ^]^ This is supported by the excited‐state TDDFT, where the first acceptor state for the 400 nm excitation is a ^1^MLCT. The longest component can be assigned to the lifetime of the relaxed ^3^MLCT state,^[^ ^7^, ^37^, ^39^, ^40^ ^]^ for which a lifetime of 12.8 ± 1.2 ps is obtained. For the constituting photosensitizer [Fe─BL], a value of 11.1 ± 0.4 ps is found (Figure S2.6; Section S2, Supporting Information). The slightly increased ^3^MLCT lifetime in [Fe─BL─Co] can be interpreted as an indirect signature of CT processes, leaking into the relaxation channel over the ^3^MLCT state. It agrees with the fact that the ^3^MLCT state in [Fe─Bl] is stabilized upon the formation of [Fe─Bl─Co].^[^ ^2^ ^]^ Yet, it does not provide unequivocal proof for a Fe → Co charge transfer due to a lack of direct spectroscopic signatures for altered charge densities at the Co cobalt center. This gap can be closed by XES.

### 2.3 Fe Kα XES Dynamics

**Figure 2:**
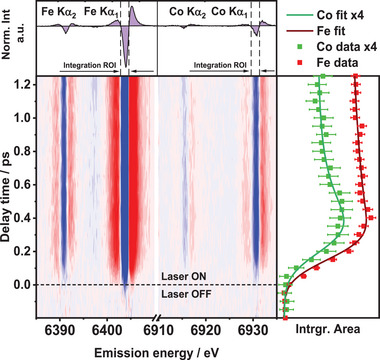
Fe and Co Kα1,2 transient XES line intensities of [Fe─BL─Co] for delay times of ‐0.2 – 1.25 ps. Top panel: transient XES signals at 1 ps delay time with integration regions of interest (ROIs) marked by vertical dashed lines. Right panel: integrated area under transient XES Fe Kα1 and Co Kα1 main feature in function of delay time (points) with corresponding fitted model (lines, top). Data for Co Kα1 were scaled by factor of 4.

Complementary to optical spectroscopy, XES offers additional sensitivity to core electrons. X‐ray emission spectroscopy (XES) is governed by different selection rules than optical absorption. The XES signal originates from localized core electrons, and through the width of the Kα_1_ XES line, it is directly proportional to the effective number of unpaired *d*‐electrons^[^ ^41^ ^]^ and ligand‐to‐metal back bonding (covalency).^[^ ^42^ ^]^ Using a multi‐crystal von Hamos emission spectrometer^[^ ^43^ ^]^ in a 2C‐XES scheme,^[^ ^44^ ^]^ transient spectra at both the Fe and Co K‐edge could be collected simultaneously (Figure S3.2, Supporting Information) without any ambiguity of time‐zero on a femtosecond timescale.^[^ ^45^ ^]^ **Figure** **2** shows the early temporal evolution of the two differential XES signals and their kinetic traces, along with the selected integration ranges for both elements. The temporal evolution of the Kα_1_ lineshapes (Figure S3.3a,b, Supporting Information) and the transient shape results from the spread of underlying transitions and line shift of the Kα_1_ line.

In [Fe─BL─Co], three time constants of *τ_1,_* ***_Fe_*** *_Co_* < 0.14 ps, *τ_3,_* ***_Fe_*** *_Co_* = 10.38(40) ps, and *τ_2,_* ***_Fe_*** *_Co_* = 1.74(18) ps are obtained from fitting of the transient kinetics at the Fe Kα_1_ emission, while for [Fe─BL] *τ_1,Fe_* ≈ 0.25 ps, *τ_3,Fe_* = 8.98(27) ps and *τ_2,Fe_* = 1.71(35) ps are found (Section S3b, Supporting Information). The most notable difference is thus the increased longest lifetime τ_3_ in the dyad, which is similarly observed in TA measurements.^[^ ^6^ ^]^ The difference of 2 ps is attributed to the different sensitivity of TA and XES toward CT states – MC states are “optically silent” in the UV–vis spectral range.

Both singlet and triplet ^1/3^MLCT states of Fe compounds are expected to have near‐identical Kα XES signatures since both have a single Fe‐localized unpaired *d*‐electron.^[^ ^8^ ^]^ Moreover, since the coupling of deep *1s* core‐hole with the 3*d* manifold is weak, Kα XES has minor sensitivity to ISC inside the MLCT manifold. Regarding metal spin multiplicity, there is a significant difference between the ^3^MLCT (*S_loc_* = 1/2) and ^3^MC (*S_loc_* = 1) states. Any relaxation process involving either of these states to the singlet ground state is thus visible in transient Kα XES experiments. In agreement with previously reported values and the current TA results, the XES time constants are assigned in the following way: the shortest lifetime *τ_1_* in both [Fe─BL] and [Fe─BL─Co] dyad corresponds to a ^3^MLCT* → ^3^MC transition.^[^ ^46^ ^]^ Indeed, partial deactivation of the ^3^MLCT to the metal‐centered ^3^MC increases XES difference due to increasing spin multiplicity at the Fe center. The time constant *τ_2_* can be attributed to the ^3^MC state decaying into the ground state.^[^ ^47^, ^48^ ^]^ The *τ_1_* time constant value entirely agrees with reports of ^3^MLCT* → ^3^MC channels in Fe(II)‐NHC complexes.^[^ ^47^, ^48^ ^]^ Moreover, the experimental lineshape was reproduced in the best way with multiplet calculations for LS Fe^II^ → LS Fe^III^ transition, providing additional proof of the dominant MLCT character of the excitation at the Fe center (Figure S3.6 a,c and Figure. S3.7, Supporting Information). Since the lineshape does not change significantly within the measured time range, the time constants *τ_3,Fe_* = 8.98(27) ps and *τ_3,_* ***_Fe_*** *_Co_* reflect the decay of the ^3^MLCT state in [Fe─BL] and [Fe─BL─Co], respectively. Finally, TDDFT excited‐state potential energy surfaces indeed identify a ^3^MC surface that intersects both the ^1^MLCT and the ^3^MLCT close to the Franck–Condon region. The ^3^MC is identified, using a Mulliken electron–hole population analysis, as a triplet state containing both a hole and an additional electron on the Fe metal, because of the Fe(*d*_xy_/*d*_yz_/*d*_xz_) → Fe(*d*_x_^2^_‐y_^2^/*d*_z_^2^) transition (Section S1d, Supporting Information; *cf*. Figure 5).

### 2.4 Co Kα XES Dynamics

**Figure 3:**
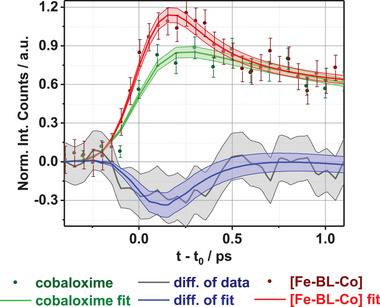
Kinetics of the Co Kα emission in [Fe─BL─Co] (red) and cobaloxime (green) after 400 nm excitation along with corresponding fits. Differential signal: [Co]‐ [Fe─BL─Co] is marked as blue lines (data+fit). Filled areas represent uncertainties.

The Co Kα transient kinetics of the dyad [Fe─BL─Co] is constituted of three time constants *τ_1,Fe_* ***_Co_*** = 0.25(1) ps, *τ_2,Fe_* ***_Co_*** = 4.12(1.39) ps and *τ_3,Fe_* ***_Co_*** ≈ 23.39 ps, a striking difference to pure cobaloxime [Co], where two time constants of *τ_1,Co_* = 2.76(31) ps and *τ_2,Co_* = 23.39(1.82) ps are found (Section S3b, Supporting Information). This difference is also evident in the (vertical offset‐corrected and area‐normalized) kinetics of the Co Kα XES in [Co] (green) and [Fe─BL─Co] (red) in **Figure** **3**, which differ substantially in shape over the first 0.5 ps (Section S3b, Supporting Information) and longer time delays (Figure S3.5a,c, Supporting Information). The difference is caused by the short decay constant *τ_1,Fe_* ***_Co_*** = 0.25(1) ps, which is not present in pure cobaloxime. This time constant thus represents a new excited state population channel occurring in the dyad, which at later timescales enhances the photocatalytic hydrogen generation and for which the optical absorption data shows Fe─Co M'MCT contribution. Consequently, the differential signal in Figure 3 is the real‐time direct signature of CT from the Fe to the Co center in [Fe─BL─Co]. A similar comparison for Fe Kα_1_ kinetic traces is in Figure S3.14 (Supporting Information).

Based on the relevant UV–vis spectrum, the direct excitation of the cobaloxime reflected in the Co kinetics of Figure 3 (green) corresponds to an LMCT state. However, the Co^III^ is in LS d^6^ the ground state with nearly degenerate e_g_ orbitals (*d*_x_^2^‐_y_^2^ and *d*_z_^2^) being empty. The [Co] transient XES signal evolves over time from symmetric around t_0_ to asymmetric for later delay times. We again recalled multiplet calculations, where the evolution of the early ΔXES signal can be reproduced with a charge transfer process, producing Co^II^, and for higher delay times with internal t_2g_– e_g_ transition accompanied by a spin flip to an LS d^6^ spin‐crossover state. Such a state would decay within 4.12(1.39) ps, a time range longer than for most SCO complexes.^[^ ^49^ ^]^ Literature suggests, that a SCO following the charge transfer is plausible, for example in Prussian‐blue analogs.^[^ ^25^ ^]^ On the other hand, according to our DFT calculations,^[^ ^2^ ^]^ the HOMO in cobaloxime is composed of degenerate *π* orbitals of the dmgH ligand, and the LUMO consists of the Co *d*_z_^2^ orbital, leading to a very weak LMCT absorption at 396 nm. The observed low cross‐section excitation could populate this LMCT state of Co in [Fe─BL─Co].

Since cobaloxime has a documented activity as a proton reduction catalyst,^[^ ^1^, ^50^ ^]^ the increased catalytic activity of [Fe─BL─Co] compared to [Fe─BL] + cobaloxime originates from the M'MCT states in [Fe─BL─Co]. Note that the signal we observe originates from an ensemble average over differently excited species since M'MCT and LMCT/SCO states cannot exist simultaneously in the same molecule. The result is evident, despite a low CT yield for our prototype dyad. However, at this point, we are unable to unambiguously determine the nature of the LMCT/SCO state. Such analysis must be supported by additional research, which is beyond the scope of the present manuscript.

### 2.5 Nuclear Motion Detected by Fe Kα XES

**Figure 4:**
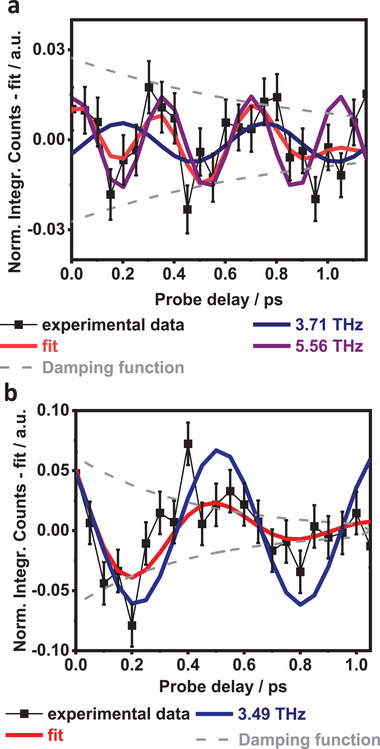
Coherent nuclear wavepacket signals (black), fitted oscillatory functions (red) part, damping (grey) for: **a)** Fe part of [Fe─BL─Co], where additionally a non‐damped parts are visible (blue, purple); **b)** same for [Fe─BL], but with only one oscillatory part (blue).

Both [Fe─BL] and [Fe─BL─Co] show distinct, coherent nuclear wavepacket signatures in the transient kinetics for iron Kα_1_. For the photosensitizer, the oscillations could be modeled by a single damped periodic function (**Figure** **4**b; Section S3d, Supporting Information), while in the case of the dyad, oscillations are composed of two contributions (Figure 4a; Section S3d, Supporting Information). [Fe─BL─Co] and [Fe─BL] share very similar half‐period oscillations, of 0.255(30) ps and 0.284(22) ps, which are shown in Figure 4a,b, respectively.^[^ ^18^, ^49^, ^50^ ^]^ Remarkably, similar oscillation periods were observed in other systems, their interpretation as coherent vibrational oscillations was recently confirmed by theory, like in [Fe(bpy)_3_]^2+^ at 580 nm excitation (0.262(10) ps),^[^ ^51^ ^]^ at 530 nm (0.265(10) ps),^[^ ^51^ ^]^ and for [Fe(bimp)_2_]^2+^ at 400 nm (0.278(2) ps).^[^ ^18^ ^]^

**Figure 5:**
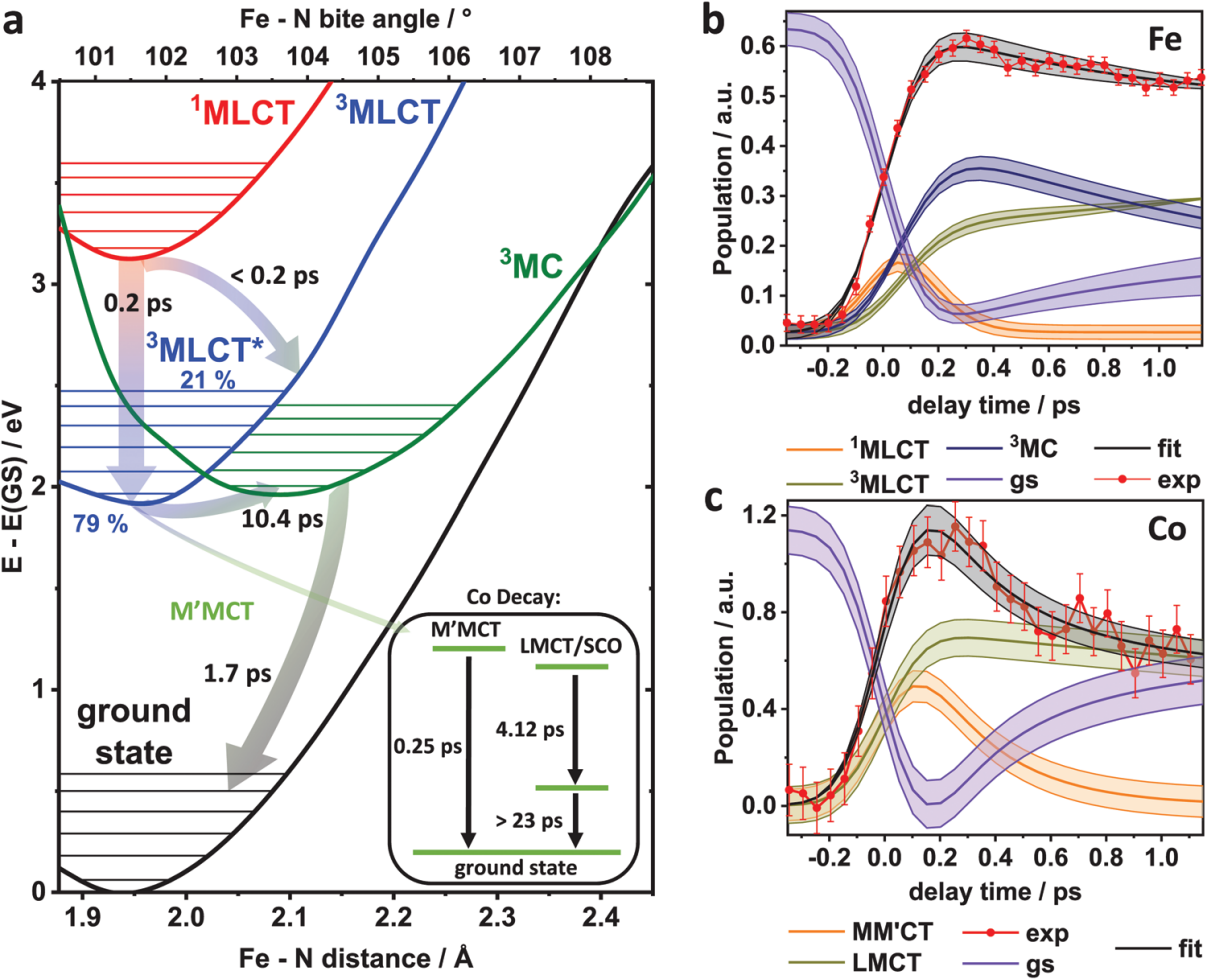
a) Ground and excited state potential energy surfaces along the Fe‐N distance (bottom x‐axis) and the Fe‐N bite angle (top x‐axis). Insert: state diagram for Co. State population analysis of **b)** Fe in [Fe─BL─Co] **c)** Co and fs‐XES signal.

An additional oscillation of 0.185(8) ps (corresponding to ≈ 180 cm^−1^) appears in the dyad as verified by the statistical F‐test (Section S3d, Supporting Information). The coherent oscillation detected in [Fe─BL─Co] (Figure 4a) is a combination of signals observed in the photosensitizer and the additional oscillation (*T_1/2_* = 0.185 ps) related to the coordination with cobaloxime. Given the previously reported findings, it is reasonable to assume that these oscillations are also a signature of coherent vibrational motion on an excited potential energy surface. Calculated excited state potential energy surfaces show that the oscillations appear along the Fe─N bonds with the equilibrium at 2.05 Å (^3^MLCT*/^3^MC crossing, **Figure** **5**a). TDDFT results indicate several vibrational frequencies in the range ≈175 cm^−1^, exhibiting a collective twisting motion of the bridging ligand, accompanied by a torsional distortion/rotation of the cobaloxime group, and slight stretching of the Fe─N bond. Raman spectra also exhibit intense bands for [Fe─Co─BL] in the 175–225 cm^−1^ range, present neither in [Fe─BL] nor in cobaloxime (Section S3d, Supporting Information). While the 0.255 ps half‐period can be associated with subsequent deactivation of the ^3^MLCT state via the ^3^MC, the 0.185 ps oscillation is likely due to one or several of the aforementioned modes which involve torsional distortion of the cobaloxime moiety and its rotation of the cobaloxime moiety and its rotation around the Fe─Co axis. This additional normal mode originates from a new structural constraint imposed by the Co moiety, thus causing structural anisotropy along the Fe─Co axis. This motion could affect the charge transfer due to the rotation of the pyridine ring and modulation of the π* orbitals overlap.

These results substantiate the excited state landscape in the [Fe─BL─Co] dyad obtained from TA spectroscopy and fs‐XES kinetics. Femtosecond XES study on [Fe(bmip)_2_]^2+^ showed excited state branching, in which a vibrational wavepacket nearly identical to the one in [Fe─BL─Co] is observed.^[^ ^18^ ^]^ A ^3^MC is partially populated from the vibrational excited ^3^MLCT* state. Since this wavepacket motion is associated with the MC state,^[^ ^24^ ^]^ it is not visible in optical TA measurements. With the minimal spectral difference between ^1^MLCT/^3^MLCT states both in TA and XES, the shortest time constant of *τ_1,Fe/_* ***_Fe_*** *_Co_* in [Fe─BL] and [Fe─BL─Co] is associated with a transition from the ^3^MLCT* to ^3^MC state. The longest time, *τ_3,Fe/_* ***_Fe_*** *_Co_* reflects the ^3^MLCT→^3^MC pathway. The remaining *τ_2,Fe/_* ***_Fe_*** *_Co_* is assigned to the ^3^MC→GS recovery.^[^ ^8^, ^21^, ^22^, ^52^, ^53^ ^]^

### 2.6 Population Analysis

Kinetic modelling further facilitates the interpretation of the obtained time constants by testing different reaction models (Section S3f, Supporting Information). At the Fe center in [Fe─BL] and [Fe─BL─Co], an additional time constant of 0.22(7) ps is obtained. According to our TDDFT calculations, this can be related to the ^1^MLCT→^3^MLCT transition after a population of the first excited ^1^MLCT state (Section S3g, Supporting Information). This short time constant includes IC and ISC.^[^ ^18^, ^21^, ^22^, ^23^, ^51^ ^]^ It also reasonably agrees with the 350 fs component obtained via TA. According to the proposed reaction scheme, the ^3^MLCT* state, which is populated during the ^1^MLCT→^3^MLCT*→^3^MLCT decay, branches into an α (^3^MLCT*→^3^MLCT→^3^MC) and β channel (^3^MLCT*→^3^MC), with contributions of 79(5) % and 21(5) %, respectively, as shown in Figure 5a. In [Fe─BL], the branching ratio is 83% to 17%, respectively (Section S3g, Supporting Information). The observed wavepacket oscillations originate from the β pathway.^[^ ^51^ ^]^

Most importantly, an additional deactivation channel, originating from the ^3^MLCT state in the form of an M'MCT electron transfer in the [Fe─BL─Co] dyad, is also confirmed by the kinetic fitting. This transfer is clearly visible when the ^3^MLCT population of the pure photosensitizer [Fe─BL] (Section S3g and Figure S3.11a, Supporting Information) and the dyad [Fe─BL─Co] (Figure 5c) in the short time window is compared. In the former, the rise of the ^3^MLCT population is initially damped due to the presence of the CT, while in the latter, the population of ^3^MLCT rises. The obtained value of the CT rate *k_et_* is very consistent with the magnitude of the differences observed for the excited state kinetics at the Co center in [Fe─BL─Co] and cobaloxime (*cf*. Figure 3).

A two‐state model with subsequent decay is used for the cobaloxime (Section S3 g and Figure S3.12,S3.13b, Supporting Information), consisting of the LMCT/SCO state directly populated upon 400 nm excitation and decaying to a lower‐level state within 2.78(3) ps. For [Fe─BL─Co] (Figure 5a), an additional electron transfer‐acceptor state (M'MCT) is compulsory from the experimental results. The M'MCT decays in 0.25 ps,^[^ ^26^ ^]^ parallel to the directly excited LMCT/SCO decay. The amplitude ratio between the direct excitation and CT transfer yield is 43.0% to 57.0%, close to the value obtained via cross‐section analysis (Section S3b and Section S3g, Supporting Information) and in line with TDDFT results. The kinetic fitting for the direct decay path requires the inclusion of an extra lowest excited state with spin multiplicity higher than the ground state and presumably having MC character.^[^ ^54^ ^]^ The lifetime of this state is estimated to be ≈23–30 ps derived from the fitting results for the pure cobaloxime. Data quality for the dyad prevents accurate fitting of this contribution to the fluorescence signal in [Fe─BL─Co].

Figure 5 a summarizes the results and conclusions from the observed time constants, literature,^[^ ^18^, ^21^, ^22^, ^40^, ^53^ ^]^ and TDDFT potential energy surfaces calculations along two reaction coordinates (Fe─ N bite angle and distances). The population analysis for [Fe─BL─Co] resulting from kinetic modeling is shown in Figure 5b,c (Section S3g, Supporting Information and the corresponding diagram for [Fe─BL]).

## 3 Conclusions

Photoactive base metal dyads appear as promising alternatives, compared to precious metals, for inexpensive and sustainable molecular assemblies capable of directly harvesting light and photocatalytic hydrogen production. This still heavily depends on the rational improvement of their performance, which involves the interplay between their molecular design and photocatalytic properties. Our study shows the tremendous potential of ultrafast 2C‐XES for direct characterization of photoinduced CT processes exemplified by the case of a noble metal‐free dyad [Fe─BL─Co] used in hydrogen production. Combined with ultrafast optical spectroscopy, multiplet calculations, TDDFT, and CASSCF/NEVPT2 calculations, and excited state modeling, we find evidence for a weak CT from the Fe^II^ photosensitizer to the cobaloxime catalyst. It contributes as a M'MCT state of 0.25 ps lifetime to the very complex excited state landscape. In addition, we can distinguish the direct excitation into an LMCT/SCO state of Co, which accompanies the CT process between both metals. The determination and visualization of the ultrafast CT is only possible by the intrinsic temporal self‐calibration of the Fe and Co Kα signals in the 2C‐XES experiment allowing unambiguous determination of the relative state positions and facilitating the data interpretation.

More importantly, building upon our results, a multitude of strategies to improve the photocatalytic activity of such base metal dyads can be deduced. It is common knowledge that the lifetime of the ^3^MLCT as the first charge‐separated state needs to be increased for iron photosensitizers to be active. From Figure 5, it is immediately clear that this is even more important here. A decreased ^3^MLCT energy would reduce the contribution of the ^3^MLCT→^3^MC decay channel, potentially in favour of the population of the M'MCT state. Another way of decreasing non‐CT decay channels would be reducing the ^3^MLCT*→^3^MC contribution. Since this pathway is connected to the nuclear wavepacket, the associated vibrational motions might play a crucial role. Further restriction of Fe─N oscillations, either via replacing N with the C atom or constructing a more rigid ligand structure, could increase the ^3^MC energy selectively. Both Fe─N and Fe─BL─Co motions are involved here according to the presented results, and substituting the pyridine with a cyclometalated ligand could be a suitable exchange for Fe─N.

The presented results thus offer a first step toward a rational design of base metal dyads for photocatalytic proton reduction reactions by direct observation and quantification of the CT process in functional bimetallic photosensitizer‐catalyst assembly by 2C‐XES.

## 4 Experimental Section

### UV‐vis Spectroscopy

The investigated complexes were dissolved in acetonitrile (spectroscopic‐grade, 2.5·10^−4^ mol L^−1^). UV–vis spectra were measured in 0.1 cm quartz cuvettes on a Lambda 465 spectrophotometer from PerkinElmer (Waltham, Massachusetts, USA). Cobaloxime (1·10^−5^ mol L^−1^) was measured with a Lambda 45 double‐beam UV spectrophotometer from Perkin Elmer (Waltham, Massachusetts, USA).

### TA Spectroscopy

The experimental setup was described elsewhere.^[^ ^6^, ^55^ ^]^ Femtosecond transient absorption dynamic studies of [Fe─BL] and [Fe─BL─Co] were conducted using a modified commercial Helios spectrometer (Ultrafast Systems, Sarasota, Florida, USA) with the IRF value of 120 fs. TA spectra were recorded for the 400 nm excitation in the 60 ps temporal range. Transient absorption measurements for selected vales of time delays were conducted across a broad range of fluences, from 13 to 52 µJ cm^−2^. The transient absorption signals in this energy density range scale linearly (Figure S2.7,S2.8, Supporting Information). Typical laser fluence was 26 µJ cm^−2^ and was of the same order as fluence of the 44 µJ cm^−2^ during X‐ray experiment. Concentrations were chosen to be identical to the time‐resolved X‐ray experiments (10 mM, MeCN), which caused high absorbance of the solutions. Therefore, optimized signal transmission was ensured by a 0.12 µm flow cell with CaF_2_ windows. Using a micro annular gear pump (≈1 ml s^−1^ flow) guaranteed the excitation of fresh solution per laser pulse and reduction of sample degradation. Subtraction of solvent response from each data set eliminated the solvent contribution in the TA data.

### Transient X‐Ray Emission Spectroscopy

Simultaneous emission of Fe and Co Kα were measured with 120 fs time resolution at the FXE instrument at the SASE1 branch of EuXFEL, Schenefeld, Germany (Figure S3.1, Supporting Information).^[^ ^45^ ^]^ The [Fe─BL─Co] dyad in a 10 mm solution of acetonitrile (MeCN) was measured in a cylindrical liquid jet (100 µm), and sample recirculation was provided by an HPLC pump. The sample was excited by 400 nm optical laser with power in the range of ≈3.4 µJ pulse^−1^ and 50 fs pulse length (FWHM = 83 µm and 34 µm for horizontal and vertical directions, respectively), which translates to ≈55% of excitation rate. The relevant fluence– signal intensity calibration is available in Figure S3.1 (Supporting Information). Electronic configuration in the ground and excited states were probed by the SASE X‐Ray beam with a central energy of 9.3 keV with 125 bunches per pulse train at 0.564 MHz intra‐train repetition rate (beam size FWHM = 20 µm, pulse duration 100 fs, ≈10^12^ photons/pulse). The X‐ray beam was operating at the standard EuXFEL mode of 10 Hz repetition rate per train, and the optical laser was at 5 Hz, meaning alternating pumped/unpumped trains. The beams were crossed with the angle of c.a. 20°. Subsequent fluorescence emission was collected using wavelength‐dispersive 16‐crystal von Hamos XES spectrometer (Fe Kα and Co Kα with Ge(440) and Si(531) analyzer crystal reflections at 75.4° and 77°, respectively) and a 2D charge integrating gain‐switching Jungfrau 1 m detector with a matrix of 1024 × 1024 pixels and repetition rate of 10 Hz. The timing jitter between X‐ray and optical pulses was ≈70 fs FWHM. The signal was integrated over 60 s (500 trains) per time point. For different delay time windows, a set of data was acquired with specified temporal step size: for 5 ps‐15 ps, it was 1 ps, while for 1.2 ps– 3.3 ps and −1.0 ps– 1.5 ps, it was 150 fs. For single delay time measurements, the signal was collected for 60s. For each measurement the number of repetitions was set individually to provide a good S/N ratio. As a reference, the catalyst cobaloxime and the photosensitizer [Fe─BL] were also measured separately in the same experimental conditions and concentrations. Due to limited solubility, cobaloxime was measured at 5 mM.

### Quantum Chemical Calculations

Unless otherwise stated, all calculations were carried out with the ORCA 5.0.1 quantum chemistry package.^[^ ^56^ ^]^ Throughout, we have used Alrich's def2‐TZVP^[^ ^57^ ^]^ basis set and employed the Split‐RI‐J method and chain of spheres (RIJCOSX) approximation to accelerate the calculation of the exchange and Coulomb terms, together with the def2/C and def2/J auxiliary bases.^[^ ^58^ ^]^ Spin‐orbit coupling corrections were introduced using the spin‐orbit mean field method.^[^ ^59^ ^]^ Solvation of the compounds was included via SMD^[^ ^60^ ^]^ (MeCN), and dispersion correction was introduced via DFT‐D3 with the Becke–Johnson damping scheme (D3BJ).^[^ ^61^, ^62^ ^]^

Unconstrained DFT optimizations of the investigated complexes were done with the PBEh‐3c method.^[^ ^63^, ^64^, ^65^ ^]^ The UV–vis spectra of [Fe─BL] and [Fe─BL─Co] were calculated using the hybrid meta‐GGA functional TPSSh,^[^ ^66^ ^]^ employing the Time‐dependent DFT (TDDFT) and the Tamm–Dancoff approximation. The adequacy of the method was justified by our benchmark study on the photosensitizer against CASSCF/NEVPT2 (Section S1a, Supporting Information). The singlet energy transitions (60 states) have been subjected to Gaussian broadening with a width of 0.2 eV (full width at half‐height) before converting to the nm scale and compared to the experimental UV–vis spectra of the investigated complexes (*cf*. SI, Section S1b,c and Figure S1.3, S1.4, Supporting Information). Donor and acceptor orbitals of selected transitions and their spatial distribution were visualized using Avogadro (*cf*. Table S1.1,S1.2, Supporting Information). Singlet and triplet excited state potential energy surfaces were computed starting from the optimized ground state geometry by discretizing a geometric pathway that involves a simultaneous stretching of the Fe─N distances at steps 0.05 Å and the Fe─N bite angles at steps of 0.7 degrees. At each point along this pathway, the 60 lowest lying singlet and triplet states were computed (i.e., 120 states in total), again using the aforementioned computational setup. To identify the nature of any given excited state, whether it is a ^1^MLCT, ^3^MLCT, or ^3^MC, we have resorted to the Mulliken population analysis coupled with an electron–hole analysis.^[^ ^67^, ^68^ ^]^ Because our TDDFT calculations are based on the singlet ground state as the reference state, the spin populations of all atoms are zero by symmetry. Instead of relying on spin densities, we identify a ^1^MLCT/ ^3^MLCT as a singlet/triplet excited state where the total Mulliken population of the Fe atom is decreased by one electron, and that of the ligand atoms is increased by one electron. A ^3^MC state is a triplet excited state where both the hole and the electron are localized on the Fe atom, corresponding to an electron transfer from the occupied *d*_xy_/*d*_yz_/*d*_xz_ orbitals to the virtual *d*_x2‐y2_/*d*_z2_ orbitals. In all cases, only excited states that lie below the initially excited ^1^MLCT were considered. Figure S1.5 (Supporting Information) depicts an example of this analysis. The geometry of the identified ^3^MC state was optimized, and its vibrational normal modes were computed in Gaussian 16 with the def2‐SVP basis set.^[^ ^69^ ^]^

## Conflict of Interest

The authors declare no conflict of interest.

## Supporting information

Supporting Information

## Data Availability

The data that support the findings of this study are available from the corresponding author upon reasonable request.
