# Supplementary material for: Ultrafast Two‐Color X‐Ray Emission Spectroscopy Reveals Excited State Landscape in a Base Metal Dyad
Source: Adv Sci (Weinh). 2024 Aug 5;11(38):2404348. doi: 10.1002/advs.202404348 (PMC11481292; doi:10.1002/advs.202404348)
Supplement: Supplementary file 1 — Supporting Information [file ADVS-11-2404348-s001.docx]

Ultrafast two-colour X-ray emission spectroscopy reveals excited state landscape in a base metal dyad

*Michal Nowakowski, Marina Huber-Gedert^,^, Hossam Elgabarty, Aleksandr Kalinko, Jacek Kubicki, Ahmet Kertmen, Natalia Lindner, Dmitry Khakhulin, Frederico A. Lim*a*, Tae-Kyu Cho*i*, Mykola Biednov,* *Lennart Schmitz, Natalia Piergies, Peter Zalden, Katerina Kubicek, Angel Rodriguez-Fernandez, Mohammad Alaraby Salem, Sophie E. Canton, Christian Bressler, Thomas D. Kühne, Wojciech Gawelda*, Matthias Bauer**

**Supplementary Information**

Table of contents:

1. Quantum chemical calculations:
   1. Benchmarking the TDDFT UV-Vis spectra of [Fe-BL]
   2. Computed and experimental UV-Vis spectrum of [Fe-BL]
   3. Computed and experimental UV-Vis spectrum of [Fe-BL-Co]
   4. Mulliken population-based electron-hole analysis of excited states
   5. Decomposing the UV-Vis spectrum of [Fe-BL] in terms of charge-transfer components
   6. Decomposing the UV-Vis spectrum of [Fe-BL-Co] in terms of charge-transfer components
2. Solvent-dependent UV-Vis data, TA experimental analysis, and fluence data
3. XES data analysis:
   1. Optical pump fluence dependence on XES signal
   2. Fluorescence fitting procedure and results
   3. A direct and non-direct contribution to Kα XES
   4. Multiplet calculations
   5. Wavepacket analysis
   6. Co Kα_1_ kinetic signals for -5-15 ps time window
   7. *d'-d* interactions in Co
   8. Kinetic model and population analysis results

**1. Quantum chemical calculations**

**a) Benchmarking the TDDFT UV-Vis spectra**

In order to understand the optical spectrum and the nature of the optical excitation process, we have resorted to quantum chemical calculations using time-dependent density functional theory (TDDFT). It is generally true that the study of transition metal complexes is challenging because of dynamic correlation effects, system size, state degeneracies or near-degeneracies, and relativistic effects on top of the typically large system sizes. In particular, TDDFT is known to have difficulties with systems having charge-transfer states, and with extended *π*-systems^1,2^, both are features of the dyad. However, despite these well-known issues, TDDFT has nevertheless been successfully applied to study such systems, including *d*^6^ transition metal complexes.^3^ These known issues mean however, that one should not blindly trust TDDFT results without scrutiny.

To this end, we have benchmarked TDDFT UV-Vis electronic spectra, using both the hybrid-GGA B3LYP functional and the hybrid-meta-GGA TPSSh functional, against CASSCF-NEVPT2 spectra. While CASSCF/NEVPT2 is known to reliably yield reasonable accuracy,^4^ the dyad molecule is too large for the method. The CASSCF/NEVPT2 method explicitly takes account of both static and dynamic correlation effects and is known to provide highly accurate spectra.^5^ In order to keep the size of the active space manageable we have done the benchmarking against the photosensitizer without the cobaloxime moiety. As explained later, the active space required to accurately compute the electronic spectrum of the photosensitizer included 14 electrons in 13 active orbitals. A CASSCF/NEVPT2 of the dyad, including all the 12 *d*-electrons together with the interacting ligand electrons was computationally unfeasible due to the large number of occupied orbitals in the active space.

Our benchmark calculations show that the TPSSh functional yields qualitatively correct result and accurately reproduces the spectrum with a slight tendency to over-estimate the frequency of the peaks, particularly the lowest-frequency peak. To obtain better comparison of calculated spectra with experimental ones calculated spectra are broadened by convolution with a Gaussian function with a width of 0.2 eV (full width at half-height), before converting the scale to nm.

- - 1. **Starting orbitals for CASSCF**

The starting orbitals for the CASSCF calculation were taken from the TPSSh ground-state Kohn-Sham orbitals at the equilibrium geometry. The TPSSh ground state has the close-lying (within 0.3 eV) Fe *d*_xy_, *d*_yz_, and *d*_xz_ orbitals as the three occupied frontier orbitals, these were naturally included in the active space. The *d*_z2_ and the *d*_x2_-_y2_ orbitals were found to be strongly mixed with ligand orbitals, consistent with the strongly σ-donating heterocyclic carbene ligand. Both the occupied (bonding) and unoccupied (antibonding) orbitals involving Fe *d*_x2-y2_ and *d*_z2_ were included in the active space. In addition to the full set of (ligand-mixed) Fe *d*-orbitals, the two highest lying occupied *π*-bonding orbitals were included in the active space, together with the four lowest unoccupied molecular orbitals (LUMO to LUMO+3). The LUMO is a *π** orbital extending over the bipyridine moiety, while the other three orbitals are all *π** orbitals extending on the CNC moieties. Thus, the final active space included 14 electrons in seven occupied orbitals and six virtual orbitals.

It is worth mentioning here that the B3LYP Kohn-Sham orbitals were identical in character to the TPSSh orbitals, in agreement with the benchmark results that we discuss below.

- - 1. **Comparison of TDDFT UV-Vis spectra to CASSCF(14,13)/NEVPT2**

The obtained spectra, which are depicted in Figure S1.1, show several interesting features. The CASSCF/NEVPT2 spectrum closely follows the experimental one, with two major peaks at 456.2 and 389.0 nm. We believe that the major source of the shift from the experimental spectrum is the implicit solvation model. Between these two major absorption peaks, there is a weak absorption peak at 422.1 nm (~10% of the oscillator strength of the strong peaks). The TDDFT spectra, although blue-shifted, still provide qualitatively correct results, except for the wrong trend in the peak intensities, with the low-frequency peak having a lower amplitude than the high-frequency one. The TPSSh functional is clearly performing better than B3LYP, with the TPSSh peaks appearing at 446.0, 415.2, and 395.9 nm, compared to 411.9, 387.1, and 365.1 nm for B3LYP. The accuracy of TDDFT transition frequencies, which we find here, is consistent with the expected accuracy range of the method, typically within 0.1-0.5 eV.^5,6^ The lack of any peaks below 300 nm in the CASSCF/NEVPT2 spectrum is because here we have only calculated the twelve lowest-lying singlet states (compared to 60 states in TDDFT).

Rather than the exact positions of the peaks, more important to our benchmark is the nature of the underlying states. Here, we find very consistent behavior between TDDFT (both functionals) and CASSCF. Both methods agree that the main transitions bear predominantly the MLCT character and originate from the three frontier orbitals to the virtual orbitals in the range LUMO – LUMO+3. The low-frequency peak is consistently the transition Fe *d*_yz_ → LUMO with contribution from the transition Fe *d*_xy_ → LUMO+2 orbital. Also, all the methods show that the higher frequency peak is mainly *d*_xy_ → LUMO+3 with a minor contribution from *d*_xy_ → LUMO+1, and that the weak intermediate frequency peak is a transition from the three frontier orbitals to the three virtual orbitals LUMO+1 – LUMO+3 (for details see Table S1.1).


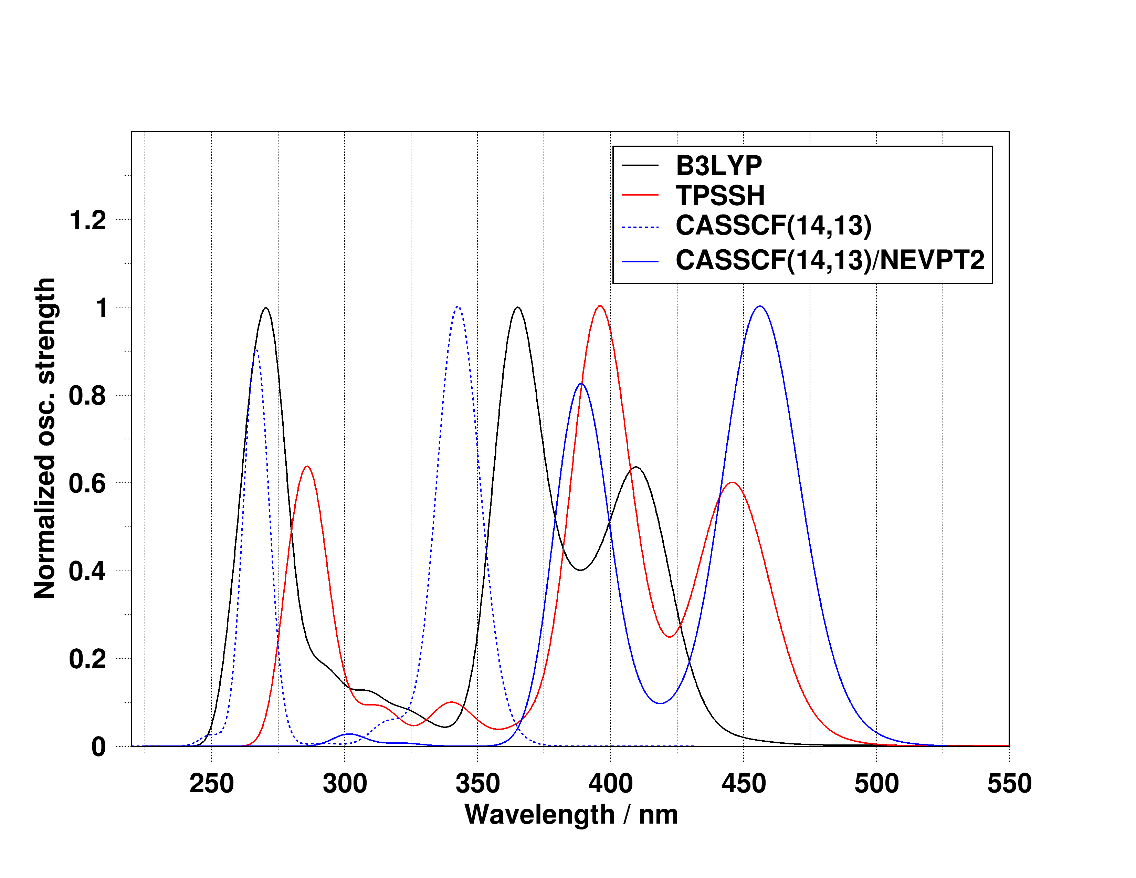


**Figure S1.1.** UV-Vis absorption spectrum of the photosensitizer in implicit acetonitrile solvation. Black: TDDFT with B3LYP, red: TDDFT with TPSSh, dotted blue: CASSCF(14,13), blue: CASSCF(14,13)/NEVPT2. All the spectra are broadened by convolution with a Gaussian function with a width of 0.2 eV (full width at half-height), before converting the scale to nm.

- - 1. **Influence of spin-orbit coupling**

In computing all the TDDFT spectra, we have included corrections due to spin-orbit coupling. It is worth noting however, that this turned out to have very little influence on peak positions, with typical shifts of less than 1 nm. As an example, Figure S1.2 shows the influence of spin-orbit coupling on the UV-Vis spectrum of the photosensitizer, as obtained with B3LYP/TDDFT.


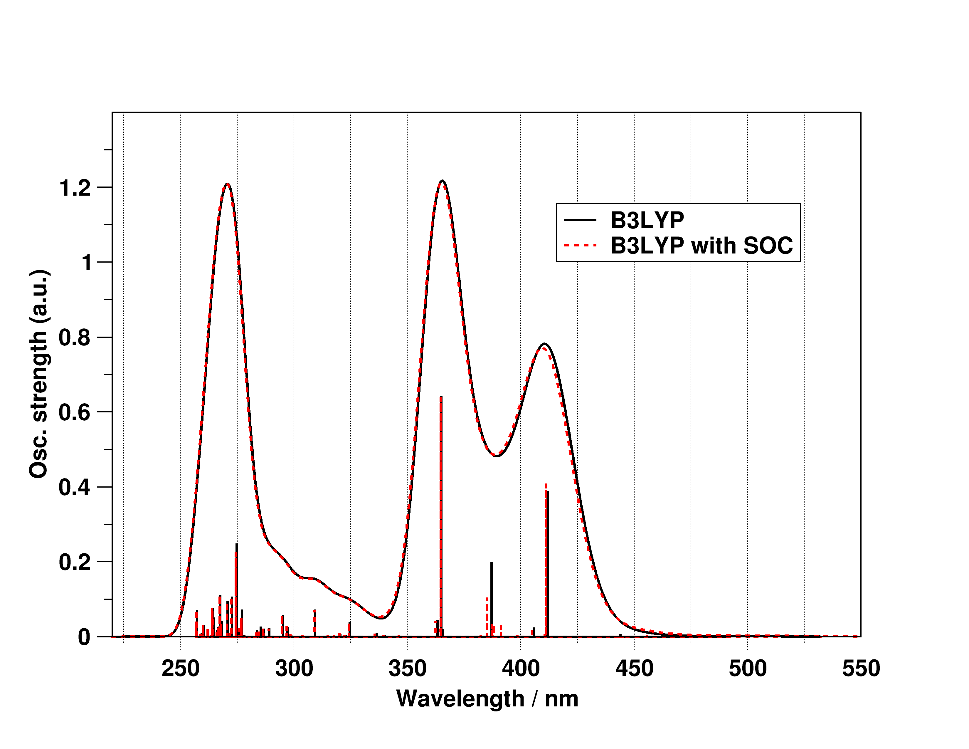


**Figure S1.2.** Influence of spin-orbit coupling (SOC) on the UV-Vis spectrum of the photosensitizer.

**b) Computed and experimental UV-Vis spectrum of [Fe-BL]**

**Figure S1.3** Experimental UV-Vis spectrum of [Fe‑BL] in MeCN and time-dependent TDDFT spectrum with TPSSh.

**Table S1.1** Computed dominant singlet vertical excitations a-c of [Fe‑BL]. Donor and acceptor orbitals are listed together with their contribution to the transition. The main character of the transition is indicated.

| Transition (state) | Donor | Acceptor | Contri-bution | Character |
| --- | --- | --- | --- | --- |
| a (8)  395.9 nm | HOMO (237)  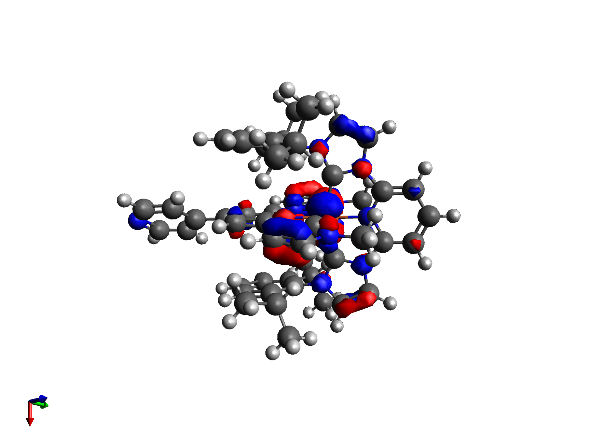 | LUMO+3 (241)  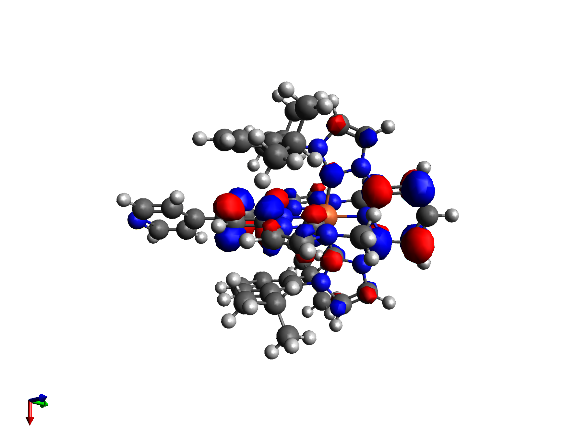 | 0.73 | MLCT |
|  | HOMO-2 (235)  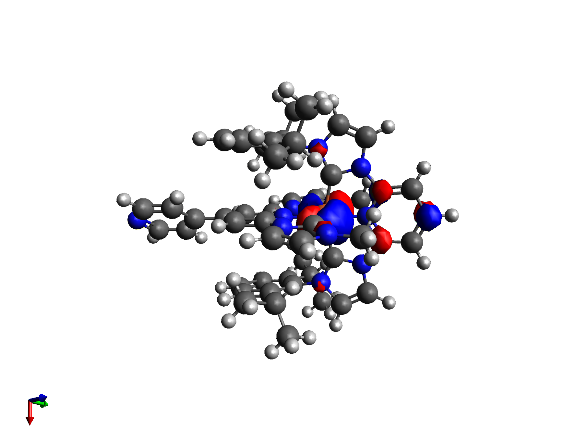 | LUMO+1 (239)  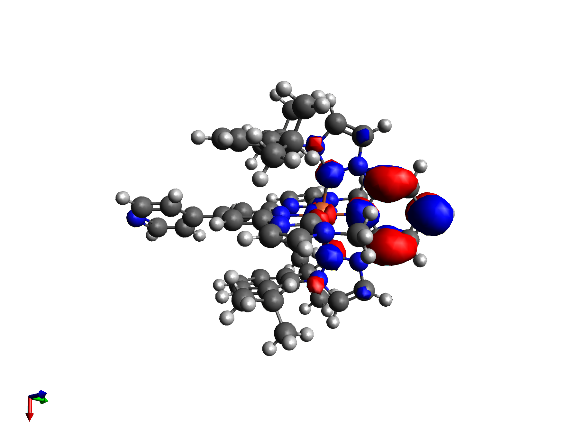 | 0.10 | MLCT |
| b (6)  415.2nm | HOMO-2 (235)  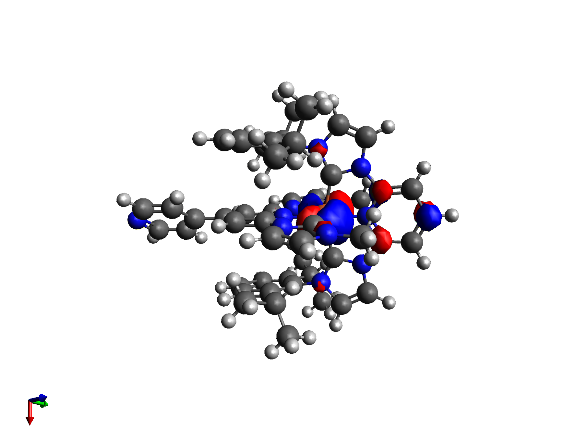 | LUMO+1 (239)  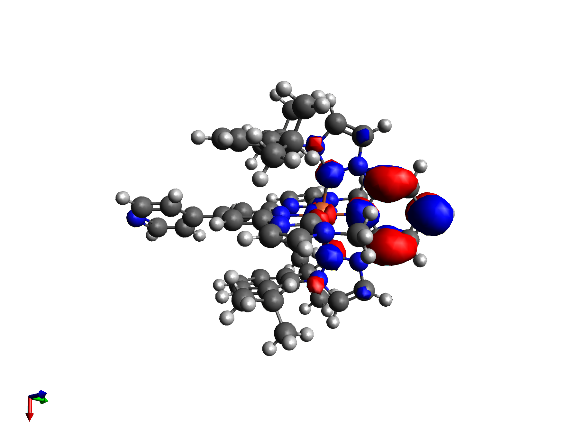 | 0.37 | MLCT |
|  | HOMO (237)  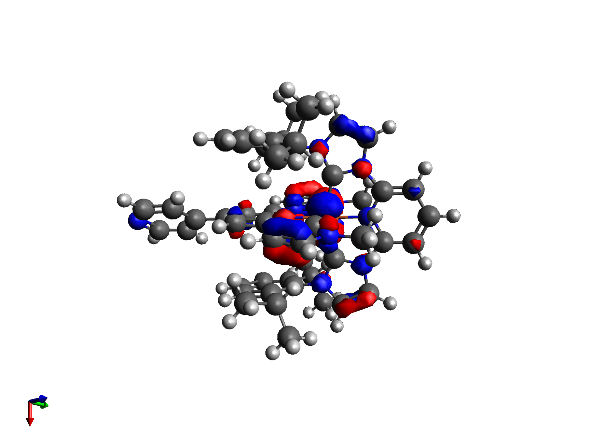 | LUMO+2 (240)  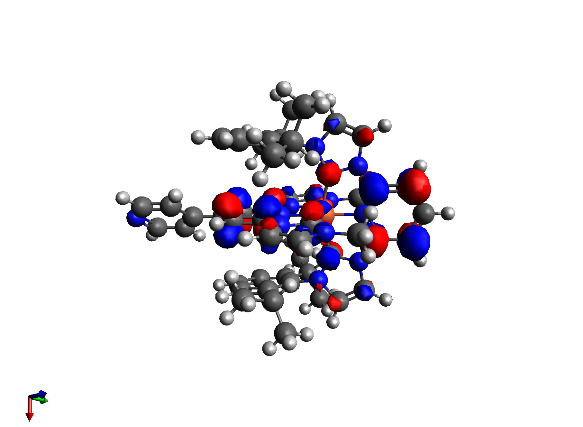 | 0.36 | MLCT |
|  | HOMO (237)  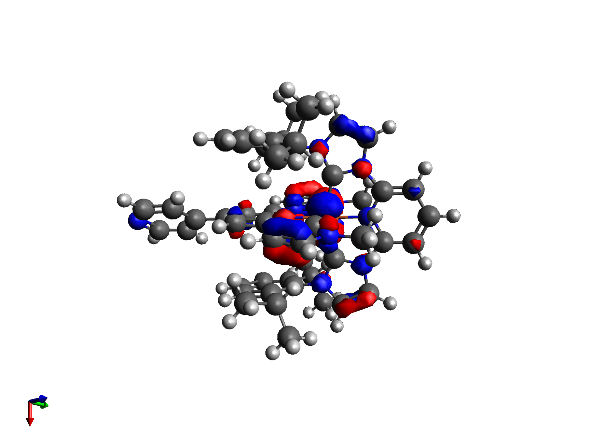 | LUMO+3 (241)  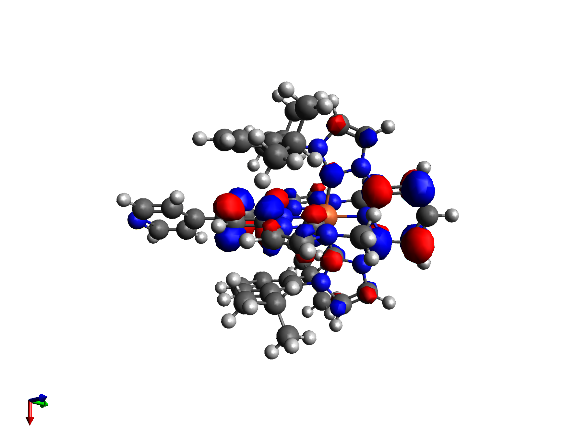 | 0.22 | MLCT |
| c (4)  446.0 nm | HOMO-1 (236)  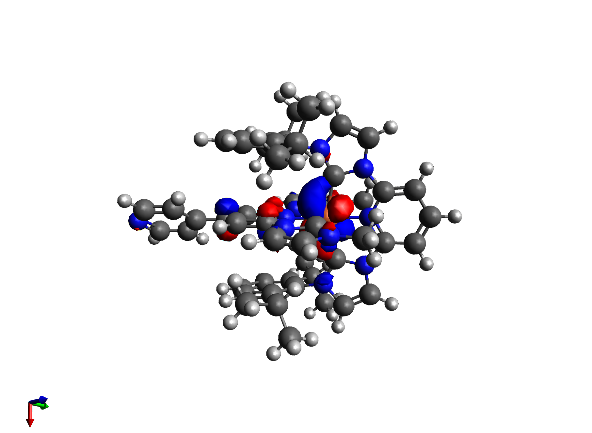 | LUMO (238)  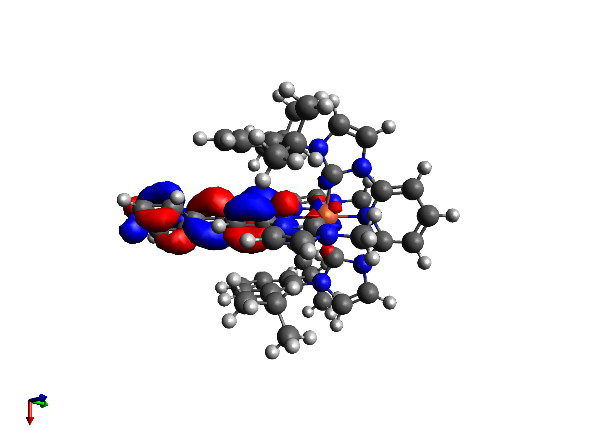 | 0.64 | MLCT |
|  | HOMO (237)  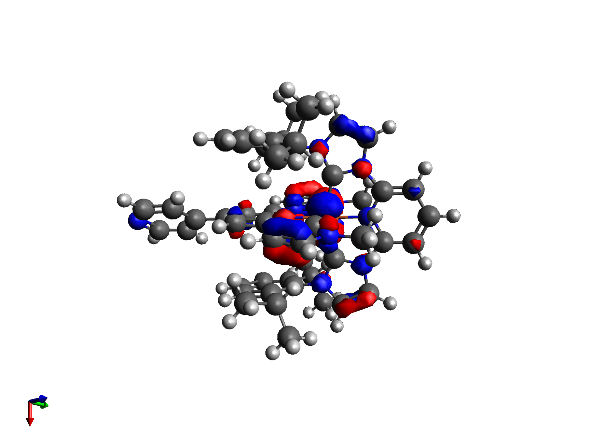 | LUMO+2 (240)  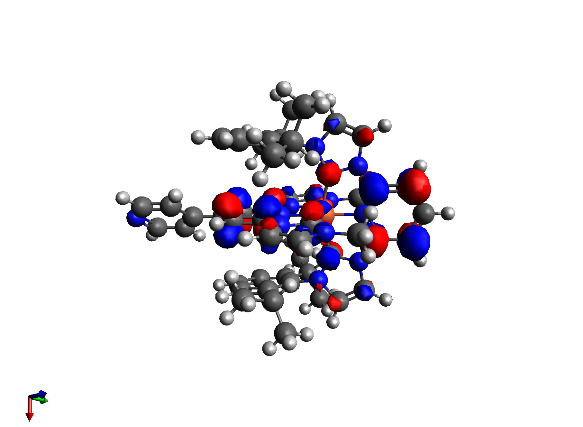 | 0.20 | MLCT |
|  | HOMO-2 (235)  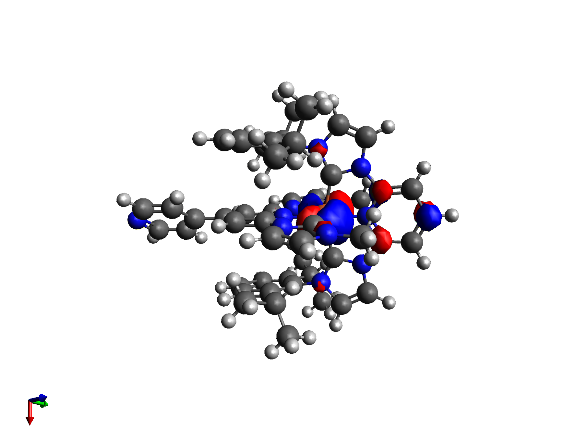 | LUMO (238)  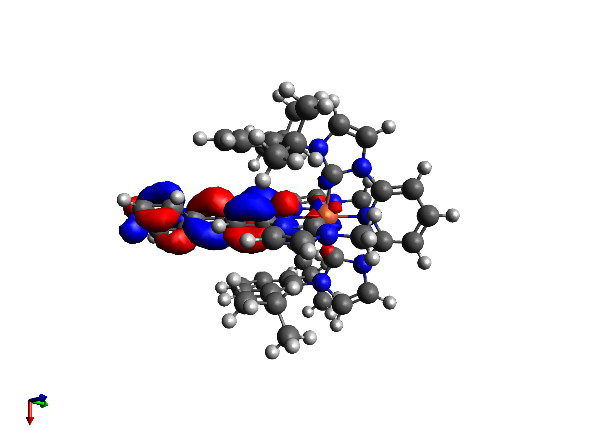 | 0.09 | MLCT |

**c) Computed and experimental UV-Vis spectrum of [Fe-BL-Co]**

**Figure S1.4** Experimental UV-Vis spectrum of [Fe‑BL‑Co] in MeCN and time-dependent TDDFT spectrum with TPSSh.

**Table S1.2** Computed dominant singlet vertical excitations a-c of [Fe‑BL‑Co]. Donor and acceptor orbitals are listed together with their contribution to the transition. The main character of the transition is indicated.

| Transition (state) | Donor | Acceptor | Contri-bution | Character |
| --- | --- | --- | --- | --- |
| a (17)  401.9 nm | HOMO (320)  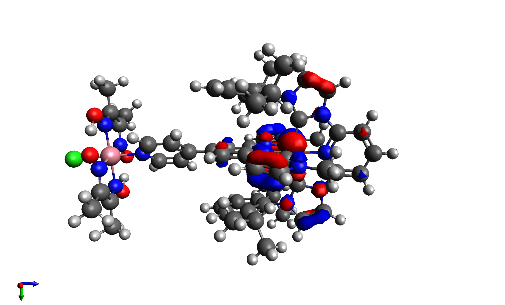 | LUMO+7 (328)  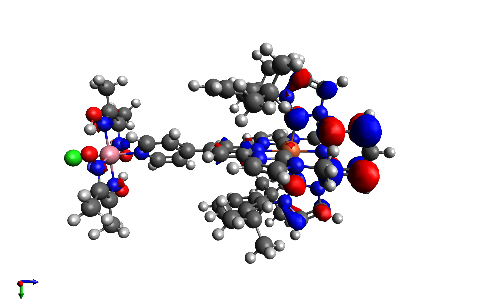 | 0.37 | MLCT |
|  | HOMO (320)  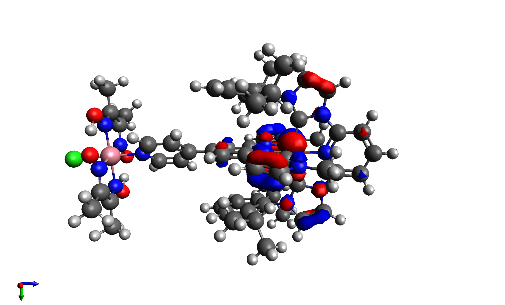 | LUMO+4 (325)  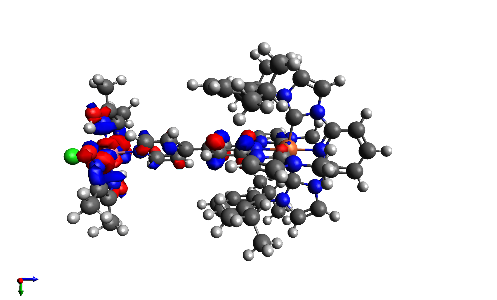 | 0.29 | MLCT/  M’MCT |
|  | HOMO (320)  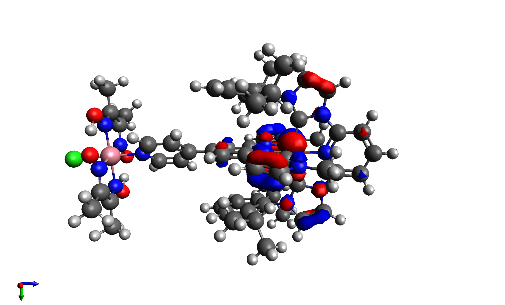 | LUMO+5 (326)  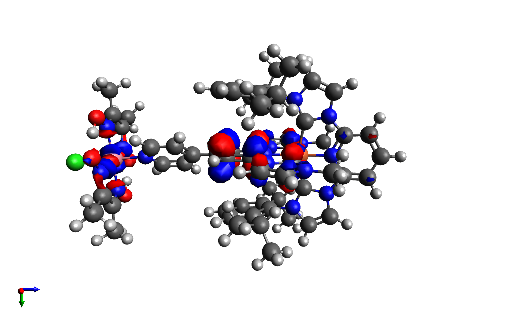 | 0.21 | MLCT/  M’MCT |
| b (15) 413.1 nm | HOMO (320)  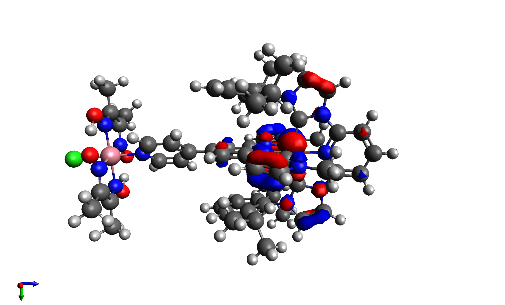 | LUMO+7 (328)  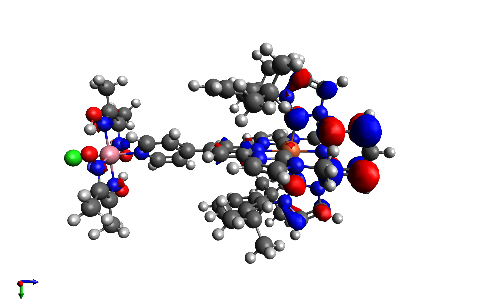 | 0.38 | MLCT |
|  | HOMO-2 (318)  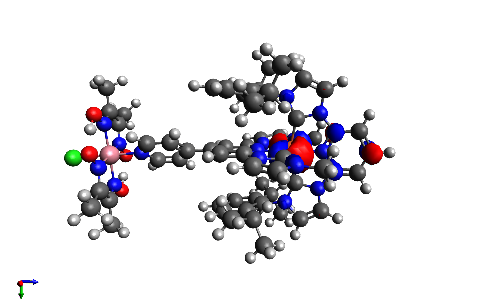 | LUMO+3 (324)  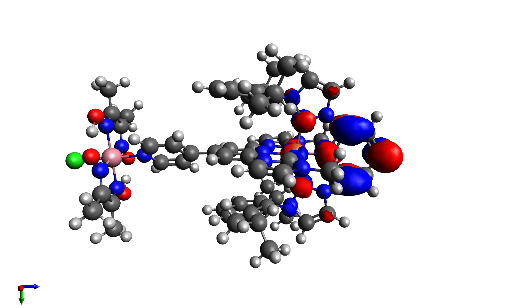 | 0.37 | MLCT |
|  | HOMO (320)  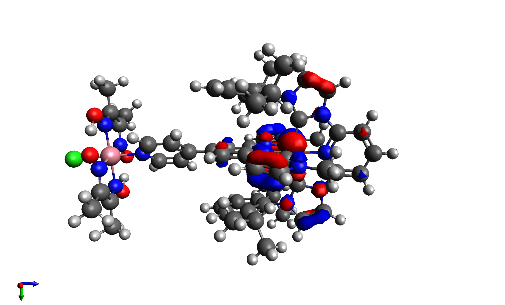 | LUMO+5 (326)  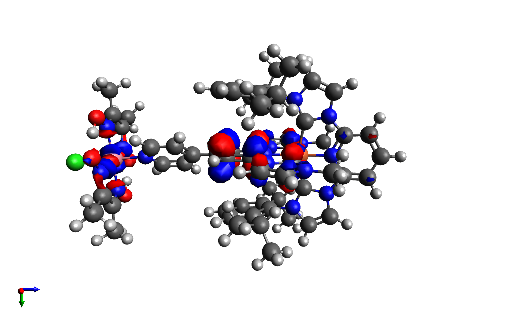 | 0.14 | MLCT/  M’MCT |
| c (8) 480.6 nm | HOMO-1 (319)  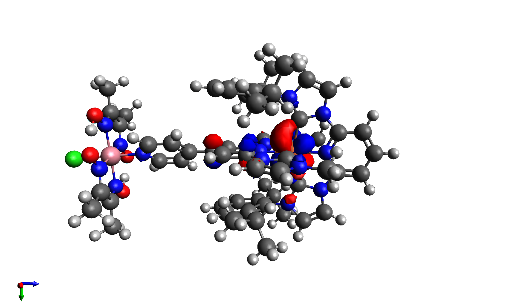 | LUMO (321)  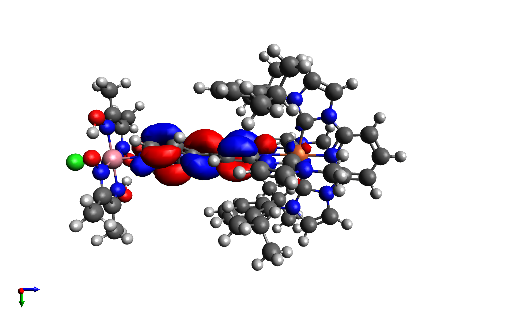 | 0.78 | MLCT |
|  | HOMO (320)  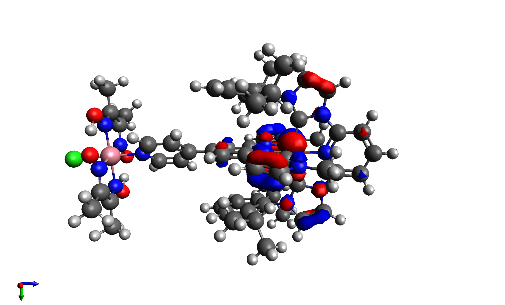 | LUMO+5 (326)  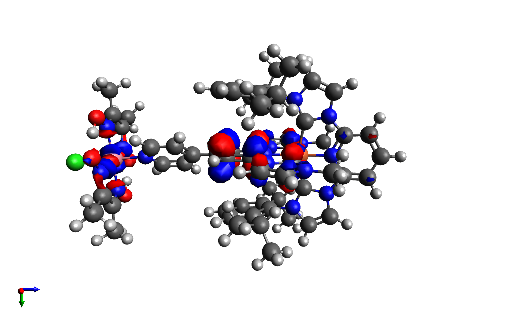 | 0.08 | MLCT/  M’MCT |

The TDDFT calculation of the dyad indicates transitions with partial M’MCT character for the UV-Vis band around 400 nm and 480 nm. The charge transfer from iron to cobalt is further analyzed by the charge transfer analysis in section 1f.

**d) Mulliken population-based electron-hole analysis of excited states**

In order to identify the nature of any given excited state as obtained from TDDFT, whether it is a ^1^MLCT, ^3^MLCT, or ^3^MC, we have resorted to the Mulliken population analysis coupled with an electron-hole analysis.^7^ Because our TDDFT calculations are based on the singlet ground state as the reference state, the spin populations of all atoms are zero by symmetry. Instead of relying on spin densities, we identify a ^1^MLCT/ ^3^MLCT as a singlet/triplet excited state where the total Mulliken population of the Fe atom is decreased by one electron, and that of the ligand atoms is increased by one electron. A ^3^MC state is a triplet excited state where both the hole and the electron are localized on the Fe atom, corresponding to a charge transfer from the occupied *d*_xy_/*d*_yz_/*d*_xz_ orbitals to the virtual *d*_x2-y2_/*d*_z2_ orbitals. In all cases, only excited states that lie below the initially excited ^1^MLCT were considered.

Figure S1.5 graphically depicts the outcome of such an analysis on the optimized geometry of the singlet ground state. In this particular case, state 7(T) is readily identified as the lowest lying ^3^MC state. Calculation of the Mulliken population contribution of the Fe atom to the hole and electron redistribution confirms the identity of this state, with the Fe atom contributing 84.8% to the electron hole (*i.e.* the excited electron originates from the Fe), and with 68% of the redistributed electron density concomitantly residing on the Fe atom (*i.e.* the excited electron resides on the Fe).

**Figure S1.5.** Mulliken population analysis of the triplet excited states that show the strongest metal-centered character. State number 53 is the initially populated ^1^MLCT state. "Fe" and "Co" refer to the Mulliken populations of the two metal atoms, "pyridine" is the total charge on the bridge pyridine attached to the Fe, and "Fe-coord" is the octahedral coordination.

- - 1. **e) Decomposing the UV-Vis spectrum of [Fe-BL] in terms of charge-transfer
        components**

This qualitative characterization of the MLCT charge-transfer nature of the main transitions in the spectrum like the one in Figure S1.1 can be put into more quantitative terms using a hole-electron analysis.^7,8^ The idea here is to start with the usual expression for the UV-Vis spectrum as obtained via broadening the excitation energies of all excited states:

$$\varepsilon\left( E \right)\propto\sum_{i} f_{i}G\left( E-E_{i}^{exc.} \right)$$

Where $E_{i}^{exc.}$ is an excitation energy, $f_{i}$ the corresponding oscillator strength, and *G(…)* denotes convolution with a lineshape function (A Gaussian function in this work). If we now subdivided the molecule into two mutually exclusive fragments A and B (generalization to more fragments is trivial), then the excitation spectrum can be readily decomposed as:

$$\varepsilon\left( E \right)_{A,B}\propto\sum_{i} f_{i}Q_{i}^{A,B}G\left( E-E_{i}^{exc.} \right)$$

where $Q_{i}^{A,B}$ is the amount of charge transfer from A to B in excited state *i* as obtained, in this case, by a Mulliken population analysis. Because the sum of all inter- and intra-fragment charge transfer terms is unity, the partitioning is exact, and the total spectrum is exactly divided into two intra-fragment (A→ A and B→ B) charge redistribution terms and two inter-fragment (A→ B and B→ A) charge transfer terms.

To decompose the photosensitizer spectrum in Figure S1.6, the structure was subdivided into three fragments: the iron atom (fragment 1), the terminal bipyridine moiety (fragment 3), and the rest of the molecule (fragment 2). Figure S1.6 depicts the decomposed TPSSh/TDDFT spectrum. The decomposed spectrum clearly reveals the nature of all the peaks in the spectrum. For instance, the low-frequency peak involves mainly (50% of the total amplitude) a charge transfer from the iron to the terminal bipyridine, where the LUMO orbital resides, but also includes important 1→ 2 and 1→ 3 charge-transfer contributions. On the other hand, the 1→ 2 charge transfer spectrum is most prominent in the peak close to 400 nm, but also the shoulder due to the contribution of the weak intermediate peak centered at 415.2 nm is also clear.


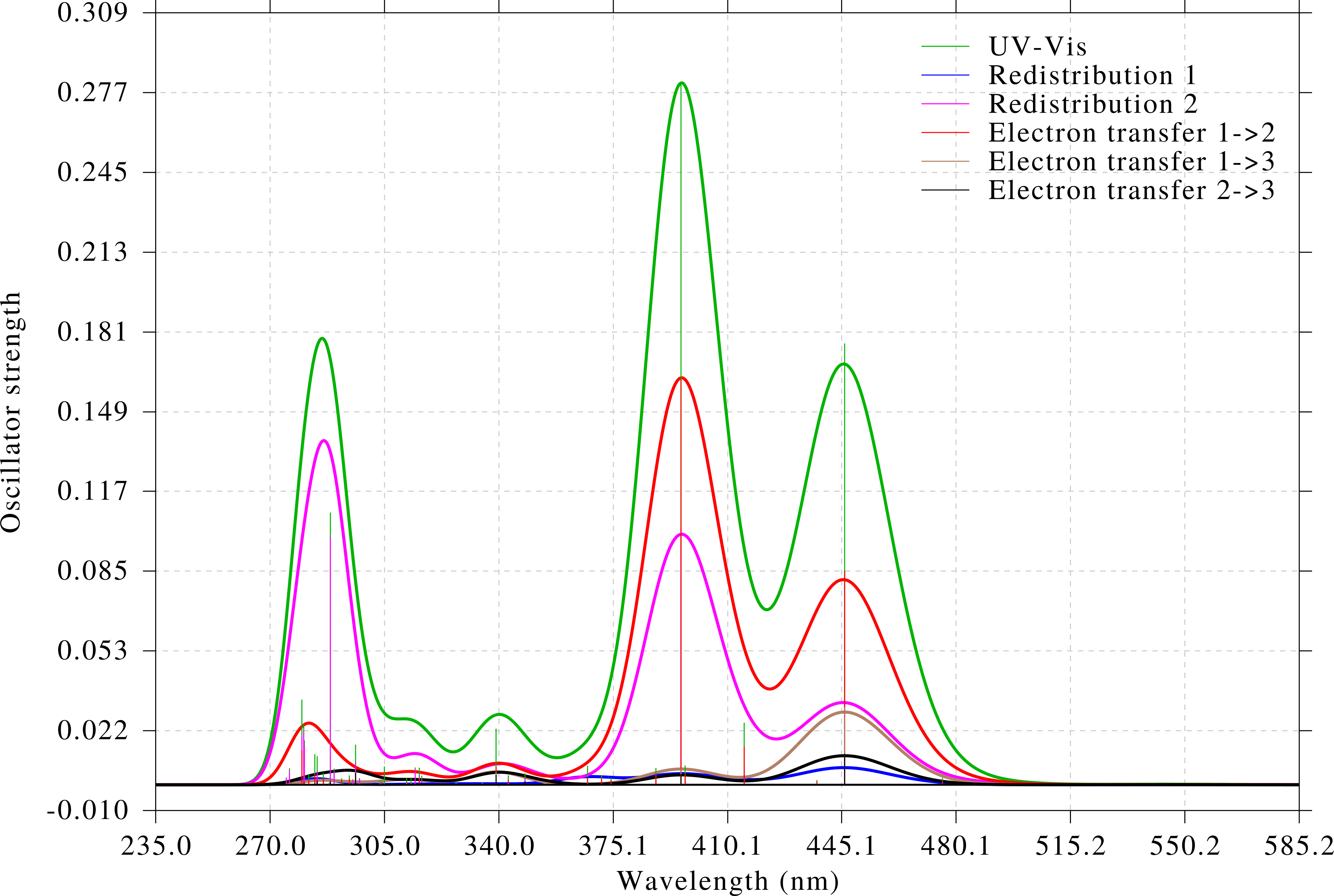


**Figure S1.6.** Decomposition of total UV-Vis spectrum into intrafragment charge redistribution and interfragment charge transfer contributions. Fragment 1 is the iron atom, fragment 3 is the terminal pyridine moiety, and fragment 2 is the rest of the molecule.

- - 1. **f) Decomposing the UV-Vis spectrum of [Fe-BL-Co] in terms of charge-transfer
        components**

In an analogic way to the [Fe-BL] case, we decomposed the UV-Vis spectrum of the [Fe-BL-Co]. Figure S1.7 shows separate parts of the dyad considered in this analysis along with the color code. Table S1.3 presents contributions to the total charge for all considered transitions for each of the molecular parts shown in Figure S1.7.


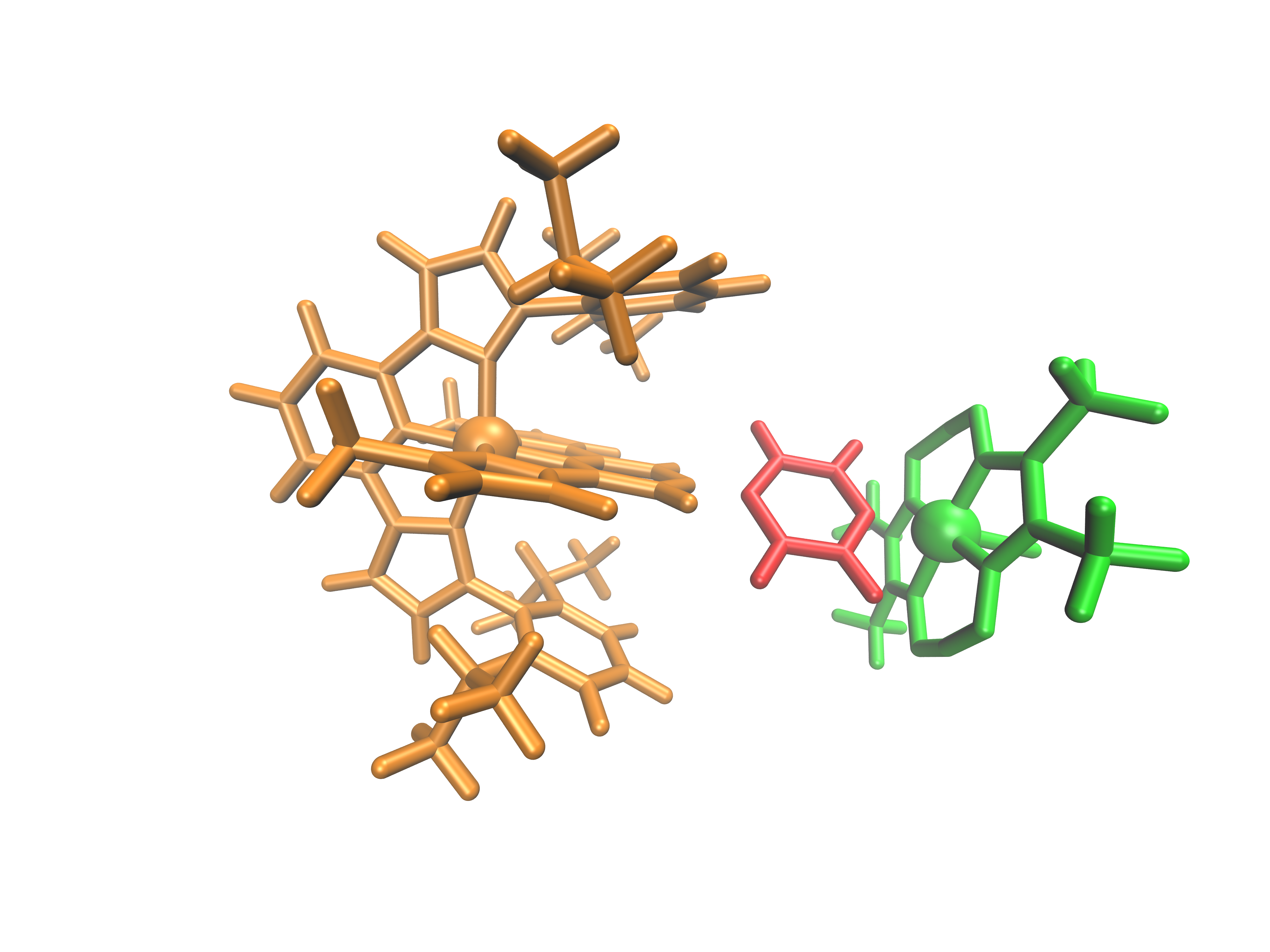


**Figure S1.7.** Definition of the three fragments used to decompose the UV-Vis spectrum. Orange: fragment 1, red: fragment 2, green: fragment 3

**Table S1.3.** The fractional contribution of each fragment to the hole and the electron in each of the three major transitions in the spectrum.

| **Wavelength / nm** | **oscillator strength** | **hole(1)** | **electron(1)** | **hole(2)** | **electron(2)** | **hole(3)** | **electron(3)** |
| --- | --- | --- | --- | --- | --- | --- | --- |
| 480.6 | 0.2949 | 0.976 | 0.560 | 0.016 | 0.414 | 0.008 | 0.026 |
| 413.1 | 0.0338 | 1.000 | 0.989 | 0.000 | 0.010 | 0.000 | 0.000 |
| 401.9 | 0.2259 | 0.993 | 0.898 | 0.002 | 0.073 | 0.006 | 0.029 |

Figure S1.8 presents the charge transfer analysis. It reveals that the peak at ~400 nm has the same nature as in the photosensitizer, with a small fraction of Fe$\to$ Co charge transfer (shown directly in Figure 1e). The low-frequency peak corresponds to considerably more charge transfer to the terminal pyridine ring.


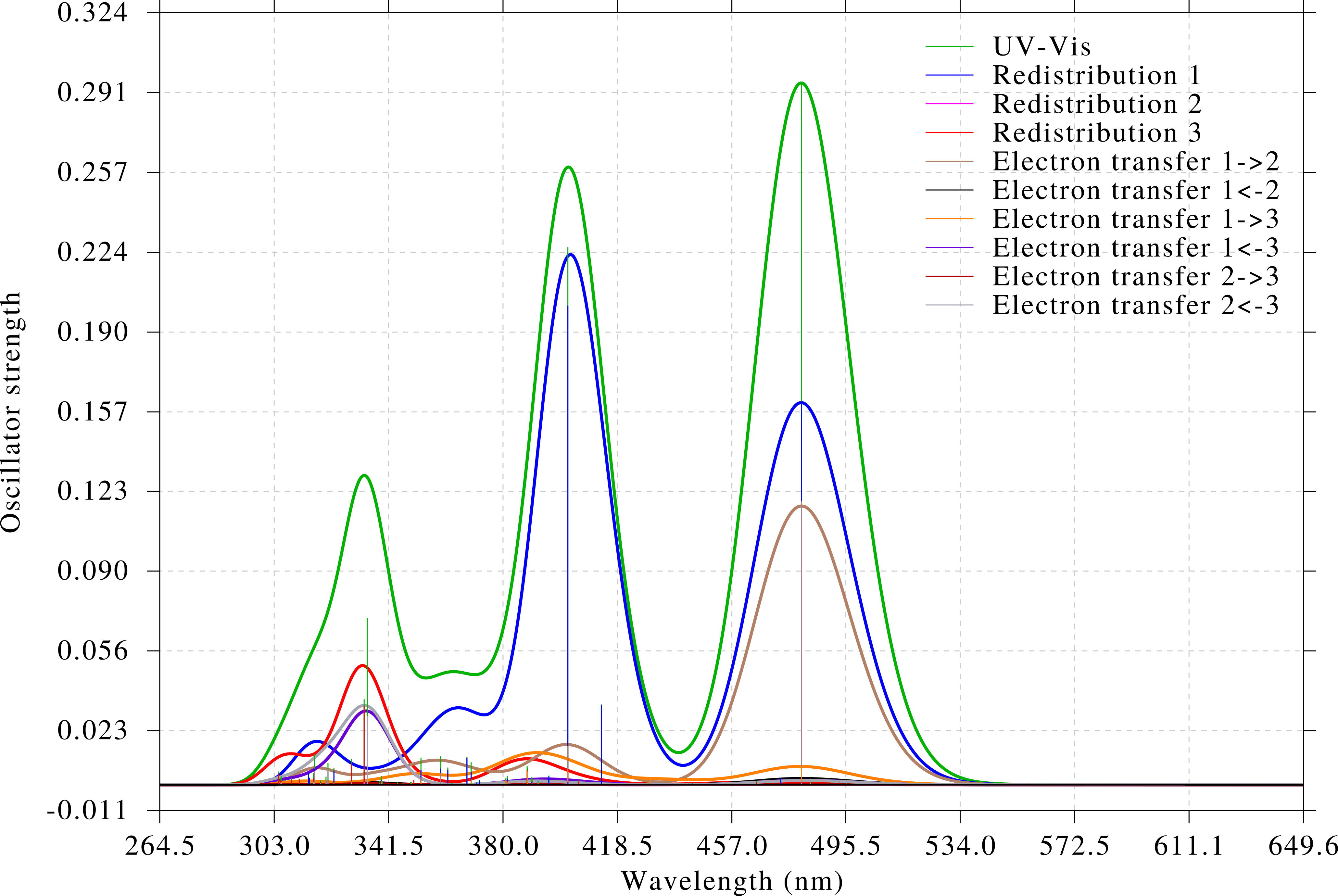


**Figure S1.8.** Decomposition of total UV-Vis spectrum into intrafragment charge redistribution and interfragment charge transfer contributions. Fragment 1 is the iron atom moiety, fragment 2 is the terminal pyridine moiety, and fragment 2 is the cobalt atom moiety.

**2. Solvent-dependent UV-VIS data and TA data analysis**

To examine the possibility of CT, two more solvents of different polarity in respect to the acetonitrile (MeCN) were tested: dichloromethane (DCM) and methanol (MeOH). Their polarity indexes are 5.8 (MeCN), 5.1 (MeOH) and 3.1 (DCM) according to “Classification of the Solvent Properties of Common Liquids”, L.R. Snyder, Journal of Chromatography, 92 (1978) 223-234. The analysis was focused on the ~400 nm peak, to match the other experiments. As visible in Fig S2.1. there is a small shift of 400 nm band towards the higher energy with the growing polarity index of the solvent, proving existence of the charge transfer effect.

**Figure S2.1.** Left: Static UV-Vis spectra measured for [Fe-BL-Co] in three different solvents; Right: re-normalized peak around 400 nm to underline wavelength shift.

**Figure S2.2.** TA spectra recorded upon 400 nm excitation for the [Fe-BL].


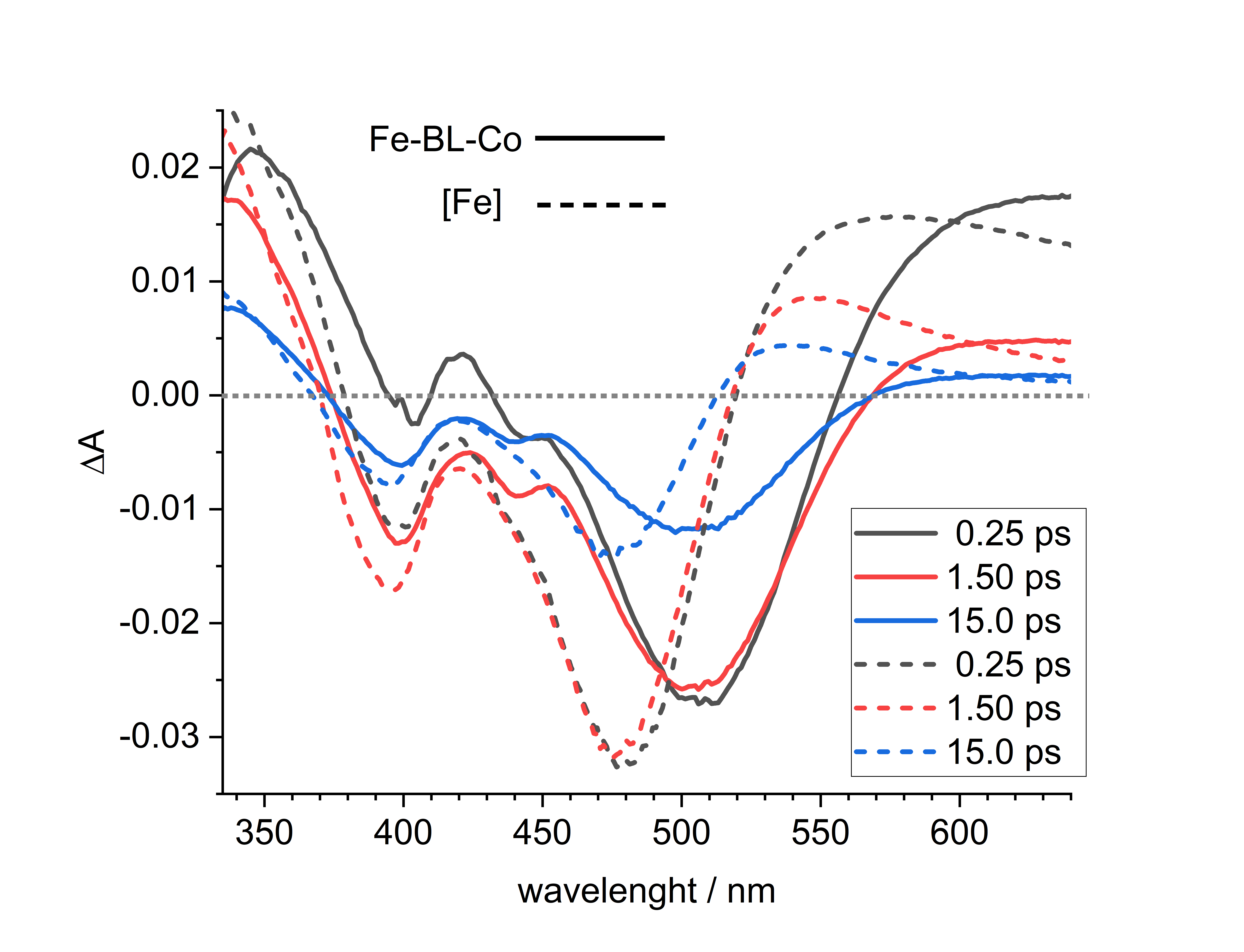
**Figure S2.3.** Comparison of transient spectra for [Fe-BL] and [Fe-BL-Co].


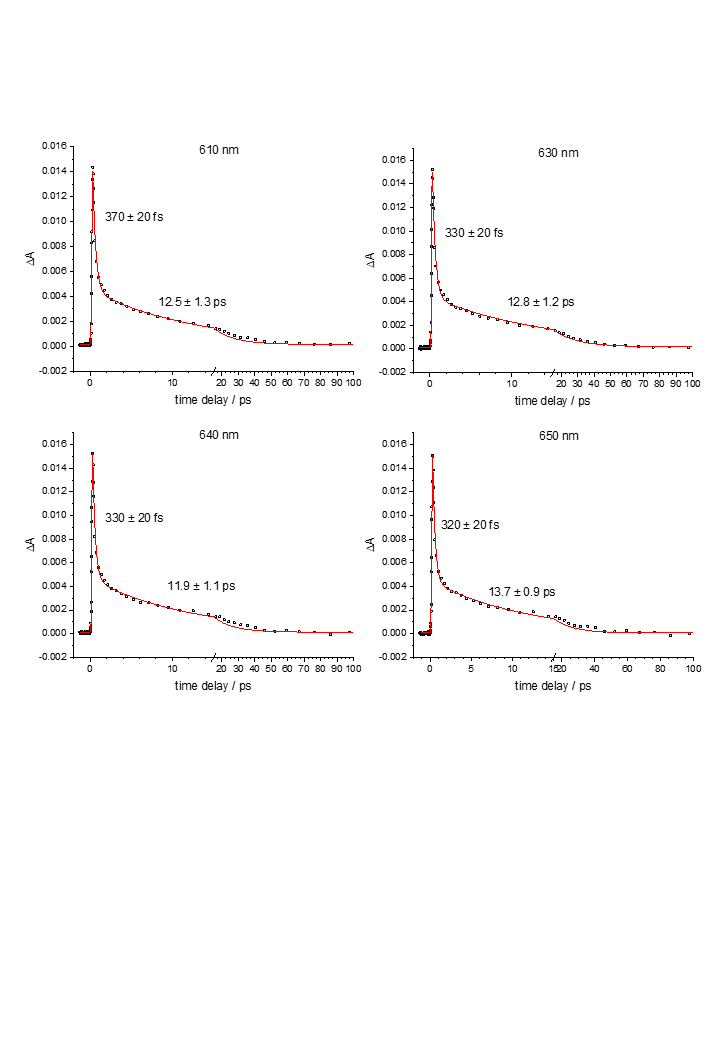


**Figure S2.4.** Kinetics recorded for [Fe-BL-Co] at 650 nm, 640 nm, 630 nm and 610 nm together with the fitted model (red lines).

**Figure S2.5.** Kinetics recorded for [Fe-BL-Co] at 510 nm and 535 nm presenting the temporal evolution of the recovery of the GS together with the fitted model (red lines).

It is commonly accepted that global analysis is applied to reveal a temporal evolution of such complexes but in this case as our model assumes that the ground state (GS) is repopulated by optically dark ^3^MC state and not directly by the ^3^MLCT (see Figure 5a) we did not use this approach. Therefore, the recovery of the GS is expected to be slower than the decay of the ^3^MLCT state, although both relaxation channels are occurring on the same order of magnitude, i.e., few tens of picoseconds. The ^3^MLCT-related time constant changes a little (11.9 ps - 13.7 ps) (Figure S2.4). Kinetics in 510-535 nm spectral range provide that the recovery of the GS takes place with a time constant in the range from 15.0 -17.0 ps depending on the wavelength selected for the strongest GS bleach band (Figure S2.5). It is very likely due to the vibrational cooling of the hot GS.^9^ Few picosecond longer recovery of GS than the decay of ^3^MLCT state is consistent with the fact that the lifetime of ^3^MC state is of the order of 2 ps (vide infra).^10–14^ The same effect can be observed in kinetics extracted for the [Fe-BL] 630 nm, 550 nm, and 480 nm (Figure S2.6). The comparison with the kinetic data between the photosensitizer and the dyad can be done only qualitatively since upon formation of the bimetallic assembly the 470 nm feature in [Fe-BL] UV-Vis spectrum relaxes to ~500 nm.

**Figure S2.6.** Kinetics recorded for [Fe-BL] at 630 nm, 550 nm and 480 nm together with the fitted model (red lines).


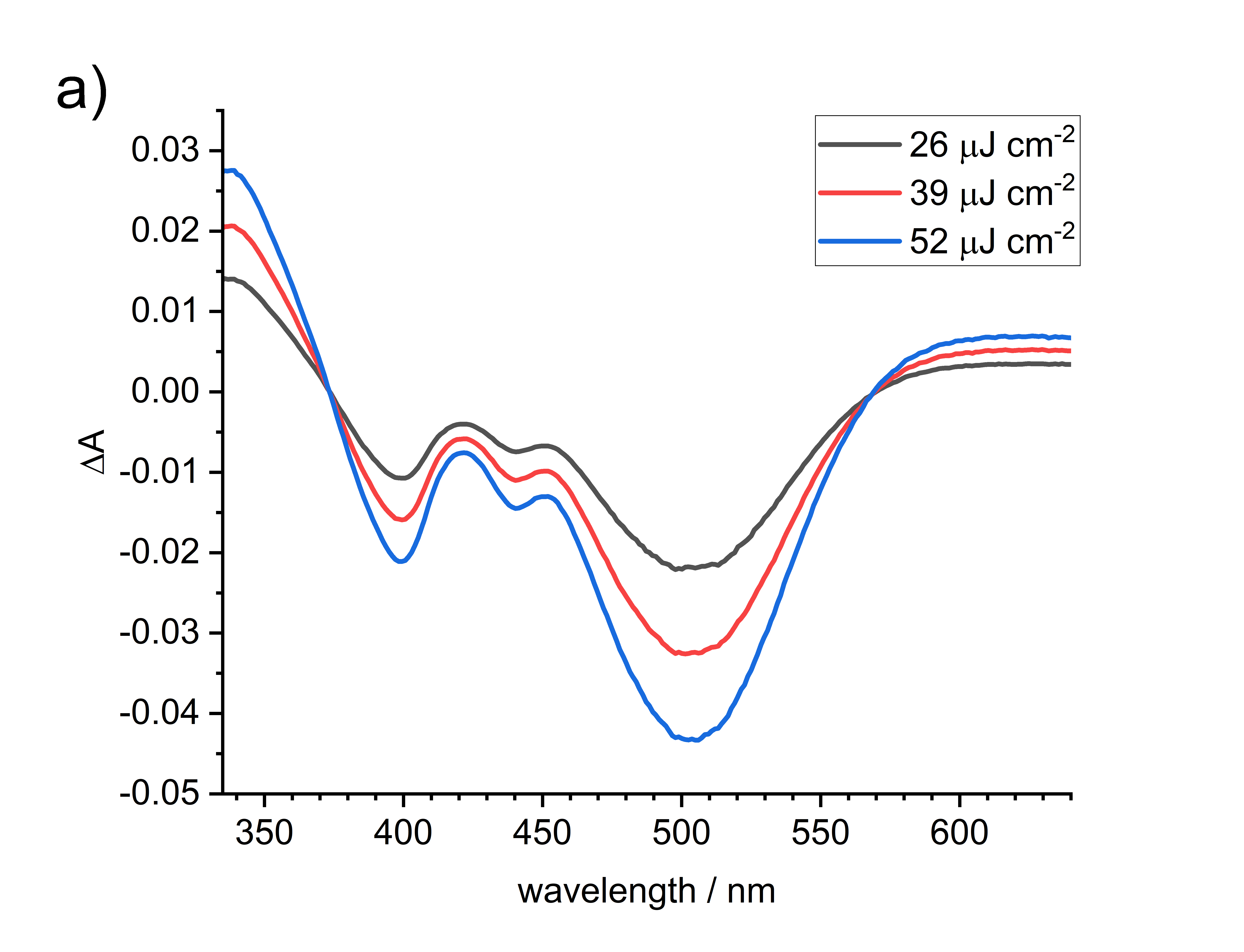

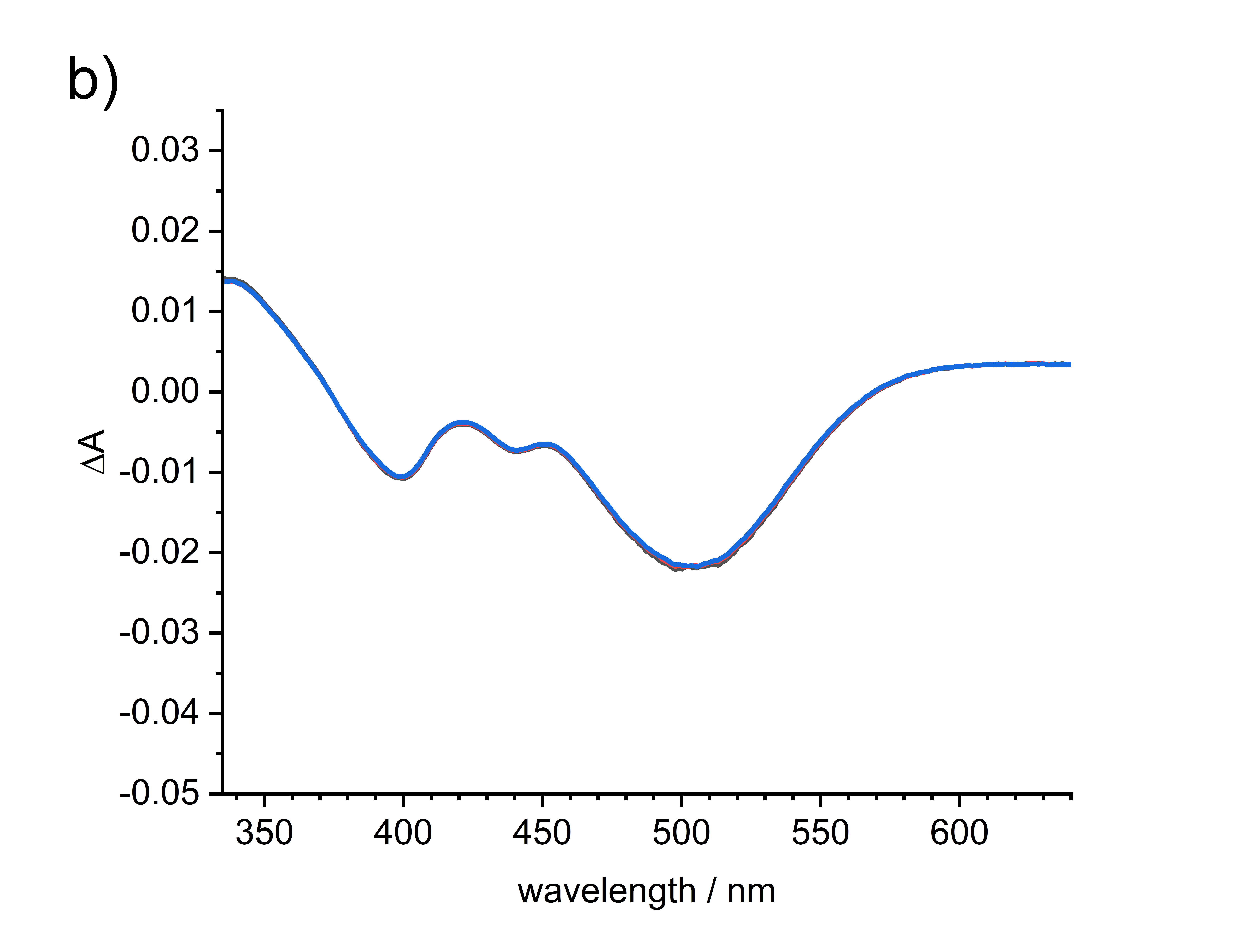


**Figure S2.7.** (a) TA signals for [Fe-BL-Co] at 5 ps time delay at different energy fluencies. (b) TA signals for [Fe-BL-Co] at 5 ps time delay normalized to the same energy fluency (26 μJ cm^-2^). Excitation at 400 nm, 10 mM solution of [Fe-BL-Co] in acetonitrile.

**Figure S.2.8.** (a) TA signals for [Fe-BL] at 5 ps time delay at different energy fluencies. (b) TA signals for [Fe-BL] at 5 ps time delay normalized to the same energy fluency (13 μJ cm^-2^). Excitation at 400 nm, 10 mM solution of [Fe-BL] in acetonitrile.


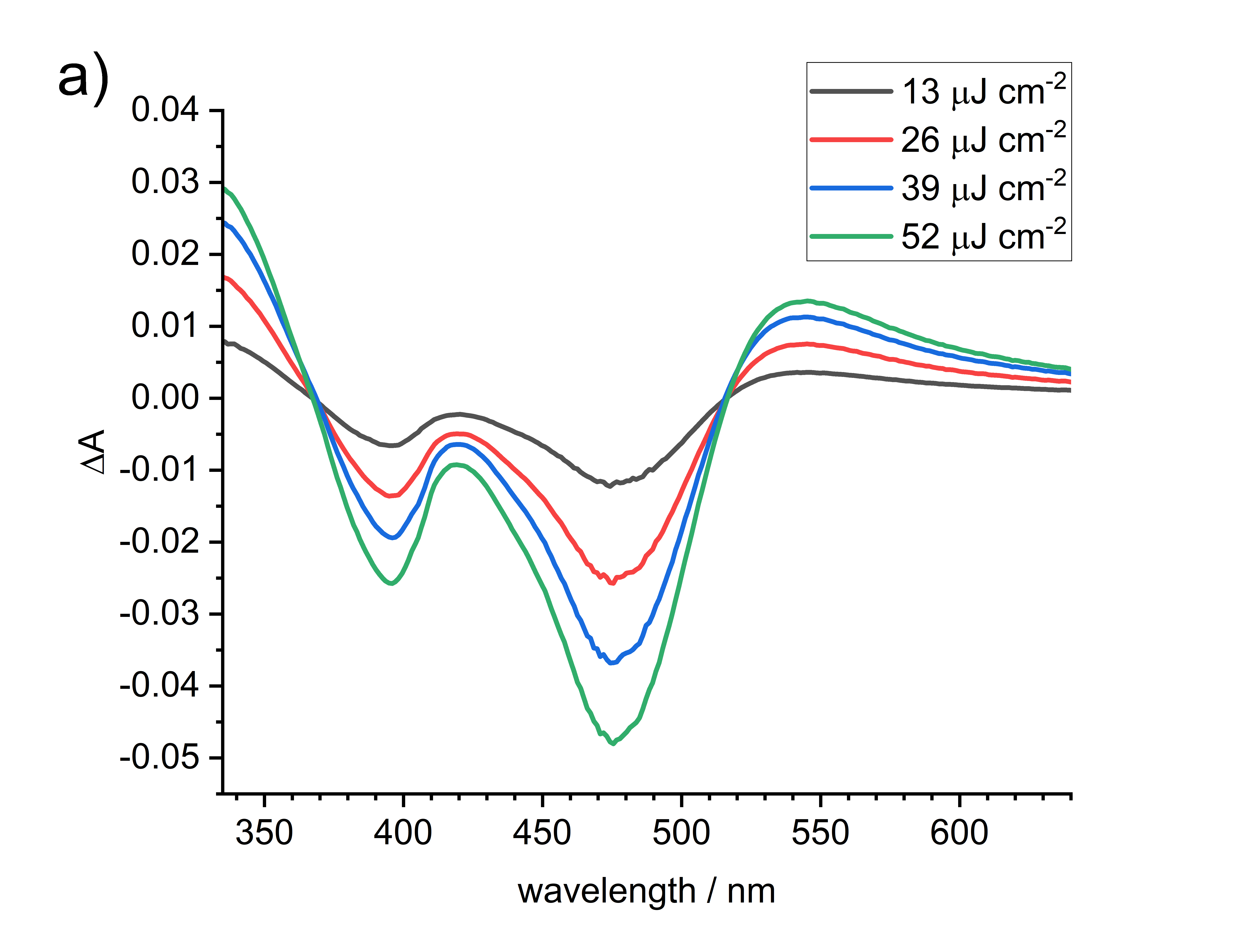

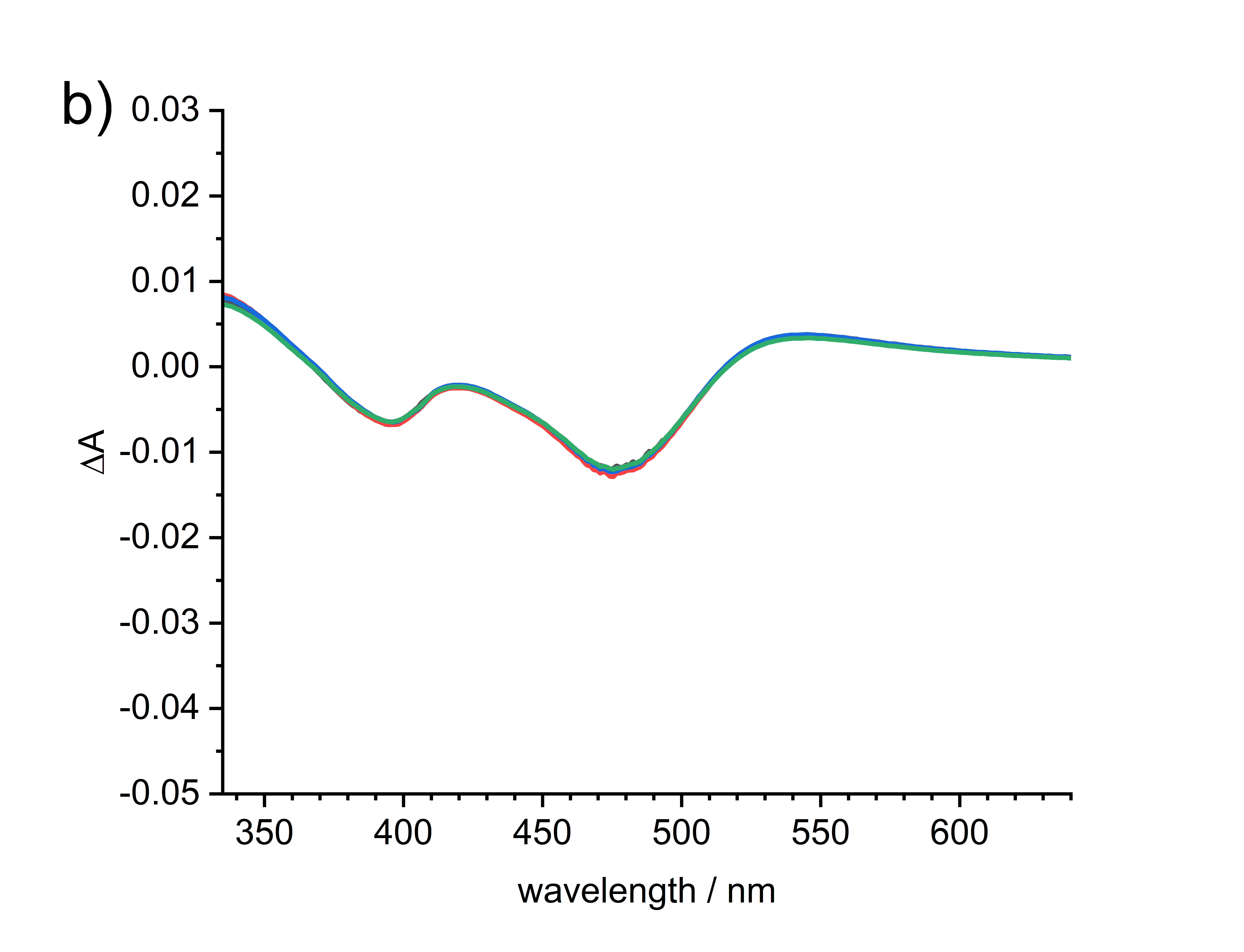


**3. XES data analysis**

1. **Optical pump fluence dependence on XES signal**

**
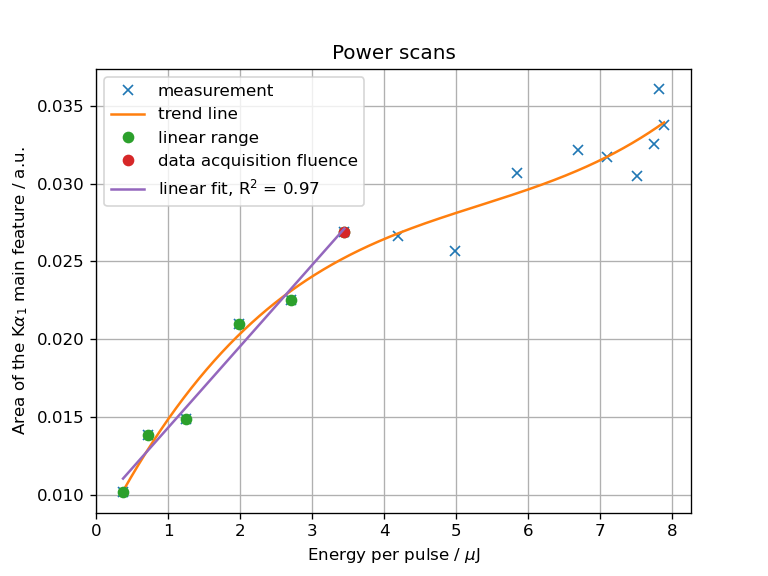
**

**Fig. S3.1** Relation between Kα_1_ feature area on the laser pulse power. The blue crosses represent data points, that are evolving along the trend line (orange). The green and red dots represent the power values in the linear regime, with red dot marking the power used in the experiment (3.4 μJ/pulse). The violet line shows the linear function fitted to the green+red data points with R = 0.97.

1. **Fluorescence fitting procedure and results**

The time-resolved X-ray emission spectroscopy (TR-XES) data was obtained on a setup scheme presented in Figure S3.2.


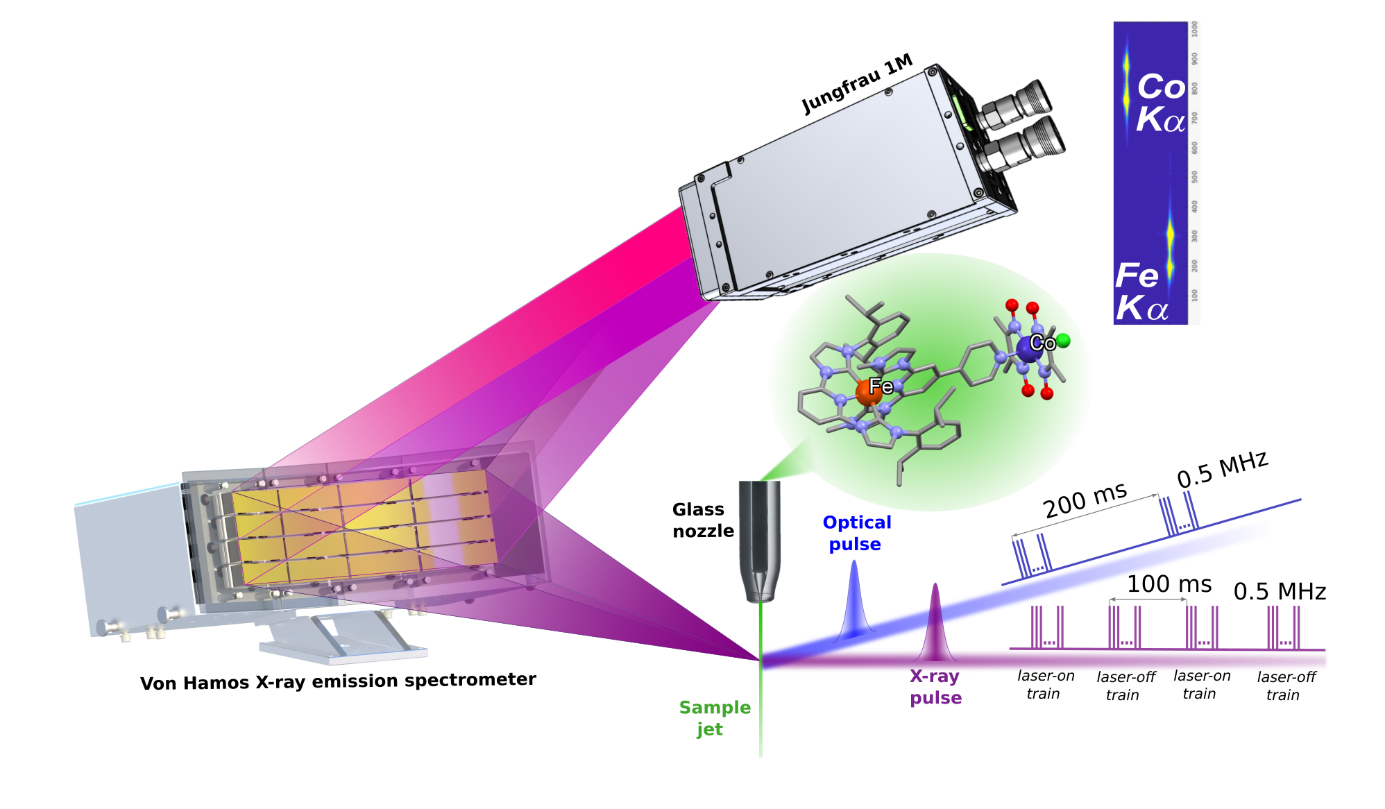


**Figure S3.2.** Scheme of the experimental setup used at FXE beamline. 50 fs FWHM optical pulses of 400 nm wavelength (blue) were synchronized with 100 fs FWHM X-ray pulses (purple) with the timing jitter of ~70 fs. Fe and Co Kα X-ray fluorescence emitted from the liquid jet (green) sample was dispersed by a 16-crystal array of von Hamos spectrometer and redirected to the 2D Jungfrau detector.

The energy scale for TR-XES data was obtained on a basis of the ground state XES (gs-XES) measurements performed using von Hamos spectrometer at P64 beamline of Petra-3 synchrotron at DESY (Hamburg). The gs-XES energy calibration was obtained by measuring Fe foil and adjusting the first inflection point in XAS spectrum to 7112 eV. Initial data correction: empty pulse removal and dark correction were conducted on-site, while data reduction and extraction were performed remotely on DESY Maxwell server with the use of self-written Python scripts. A set of data from the experiment was sorted, background reduced, filtered, and normalized to obtain ON/OFF XES spectra in respect to delay time between the optical pump and X-ray probe pulses. From that a series of XES spectra, differential (transient spectra, ΔXES) spectra were calculated, as *ΔXES(t) = XES_ON_(t) - XES_OFF_(t),* both for Fe and Co Kα lines, examples are in Figure S3.2 a-b. To obtain pure excited state spectra (cleared ON) ground state spectra (OFF) multiplied by (1-excitation rate) factor were subtracted from ON spectra. The resulting pure ON spectra were re-normalized before corresponding transient signals were calculated (cleared transient). Example results are shown in Figure S3.3 c-d. Progressing changes in the ΔXES profile were represented in form of the integral of the selected feature over all delay times. Those kinetics were subsequently fitted with fluorescence rise and *i*–exponential decay functions to give decay rates $\tau_{i}$ ^15^:

$y=y_{0}+\sum_{i} A_{i}g_{i}\left( t \right)$ (S3.a.1)

$g_{i}\left( t \right)=\frac{1}{2}\left( 1+erf\left( \frac{t-t_{0}}{\sigma}\frac{C}{2}- \frac{\sigma}{2{C\tau}_{i}} \right) \right)e^{\frac{\sigma^{2}}{C^{2}\tau_{i}^{2}}}e^{-\frac{t-t_{0}}{\tau_{i}}}$ (S3.a.2)

where:

**Figure S3.3.** Kα transient XES at 0.45 ps, 3.0 ps and 15 ps for: **a)** Fe @ [Fe-BL-Co]; **b)** Co @ [Fe-BL-Co]. The lineshape origin and comparison of the transients obtained from a clear excited state XES spectrum (cleared ON) and directly from ON spectrum: **c)** at 0.0 ps; **d)** at 0.5 ps.

**a**

**c**

**b**

**d**

$C=2\sqrt{ln\left( 16 \right)}$ ;

$A_{i}$ – amplitude for the *i* –th exponent;

$t_{0}$ – the time-zero constant value;

$\sigma$ – Gaussian broadening due to IRF function. Due to used setup the IRF was fixed to 0.28 ps;

$i=1,2,3$ – the degree of exponential function.

The fitting procedure was carried out in two steps. First, the largest dominating contribution was fitted to a kinetic trace in the time window of 20 ps and step size of 1 ps. Second, to fit shorter decay time constants, kinetic traces in time windows of ~2 ps and step size of 50 fs with the largest time constant were fixed. All fitting procedures were performed with a value of FWHM in pump-probe cross-correlation function set to *σ =* 0.284 ps. The σ value was refined in the post-fitting verification. The summarized fitting results are presented in Table S3.1 and in Figs. S3.4-5.

**Table S3.1.** Summary for fluorescence fitting to experimental data.

| **[Fe-BL] 15 ps** | | **[Fe-BL] 1.2 ps** | |
| --- | --- | --- | --- |
| A_1_ | - | A_1_ | 0.170(26) |
| τ_1_ [ps] | - | τ_1_ [ps] | 0.245(42) |
| A_2_ | 0.079(12) | A_3_ | 0.126(8) |
| τ _2_ [ps] | 1.705(348) | τ _3_ [ps] | 2.421(640) |
| A_3_ | 0.241(5) | A_2_ | 0.538(7) |
| Τ_3_ [ps] | 8.984(273) | τ _2_ [ps] | 10.142(2.054) |
| t_0_ [ps] | 0.368(31) | t_0_ [ps] | 0.015(7) |
| $y_{0}$ | 0.070(2) | $y_{0}$ | 0.061(6) |
| FWHM^a^ [ps] | 0.289(61) | FWHM^a^ [ps] | 0.305(21) |
|  |  |  |  |
| **Fe @ [Fe-BL-Co] 15 ps** | | **Fe @ [Fe-BL-Co] 1.2 ps** | |
| A_1_ | - | A_1_ | 0.109(20) |
| τ_1_ [ps] | - | τ_1_ [ps] | 0.115(23) |
| A_2_ | - | A_2_ | 0.111(4) |
| Τ_2_ [ps] | - | τ_2_ [ps] | 1.740(182) |
| A_3_ | 0.00135(2) | A_3_ | 0.483(3) |
| τ_3_ [ps] | 10.381(242) | τ_3_ [ps] | 12.417(1.399) |
| t_0_ [ps] | -0.064(12) | t_0_ [ps] | 0.010(3) |
| $y_{0}$ | 4.922(56) ·10^-4^ | $y_{0}$ | 0.024(2) |
| FWHM^a^ [ps] | 0.284(48) | FWHM^a^ [ps] | 0.275(10) |
|  |  |  |  |
| **Co @ [Fe-BL-Co] 15 ps** | | **Co @ [Fe-BL-Co] 1.2 ps** | |
| A_1_ | 3.140 (421) ·10^-4^ | A_1_ | 0.00257(13) |
| τ_1_ [ps] | 0.25 (fixed) | τ_1_ [ps] | 0.242(14) |
| A_2_ | 1.249(175) ·10^-4^ | A_2_ | - |
| τ _2_ [ps] | 4.12(1.39) | τ _2_ [ps] | - |
| A_3_ | 1.381(149) ·10^-4^ | A_3_ | 0.00187(37) |
| τ _3_ [ps] | 23.39 (fixed) | τ _3_ [ps] | 6.084(1.134) |
| t_0_ [ps] | -0.116(10) | t_0_ [ps] | 0.047(7) |
| $y_{0}$ | 1.049(31) ·10^-4^ | $y_{0}$ | 8.061(282) ·10^-4^ |
| FWHM^a^ [ps] | 0.284(42) | FWHM^a^ [ps] | 0.280(19) |
|  |  |  |  |
| **Cobaloxime 215 ps** | | **Cobaloxime 1.2 ps** | |
| A_1_ | 2.342(233) ·10^-5^ | A_1_ | 4.011(110) ·10^-5^ |
| τ_1_ [ps] | 2.764 (312) | τ_1_ [ps] | 2.391(172) |
| A_2_ | 3.391(78) ·10^-5^ | A_2_ | - |
| τ _2_ [ps] | 23.391(1.820) | τ _2_ [ps] | - |
| t_0_ [ps] | -0.056(0.093) | t_0_ [ps] | -0.015(0.040) ·10^-2^ |
| $y_{0}$ | 4.812(51) ·10^-5^ | $y_{0}$ | 3.391(35) ·10^-5^ |
| FWHM^a^ [ps] | 0.273(50) | FWHM^a^ [ps] | 0.305(84) |

^a^ Post-fitting refinement

**Figure S3.4.** Fluorescence decay fitting results for: **a)** [Fe-BL], long-time window; **b)** [Fe-BL], short-time window; **c)** Fe @ [Fe-BL-Co], long-time window; **d)** Fe @ [Fe-BL-Co], short-time window.

**a)**

**b)**

**c)**

**d)**

**Figure S3.5.** Fluorescence decay fitting results for: **a)** [Co] @ [Fe-BL-Co], long-time window; **b) [**Co] @ [Fe-BL-Co], short-time window; **c)** cobaloxime, long-time window; **d)** cobaloxime, short-time window.

**a)**

**b)**

**c)**

**d)**

1. **A direct and non-direct contribution to Kα XES**

At the optical pump wavelength of 400 nm both Fe and Co centres were simultaneously excited (although predominantly Fe site) and probed with 9.3 keV X-rays. Hence, we have carried out rigorous and detailed analysis to distinguish the direct and non-direct contributions originating from the photoexcitation at the Co center in the studied dyad. In this case, we have computed the X-ray and optical convoluted cross-section relation *C_σ_* of cobaloxime and Co part of the dyad as follows:

$C_{\sigma}\sim\frac{\sigma_{X-Ray}^{cobaloxime}\sigma_{UV-VIS}^{cobaloxime}}{(\sigma_{X-Ray}^{dyad}-\sigma_{X-Ray}^{PS})(\sigma_{UV-VIS}^{dyad}-\sigma_{UV-VIS}^{PS})}\approx0.59$

This number can be compared to the average ratio between the intensity of cobaloxime kinetic trace and [Co] part of dyad kinetic trace, which was estimated to be 0.88. Our analysis yields that approximately 59% of Co signal in dyad originates from the different electronic structure around Co site in the dyad, as compared to isolated cobaloxime. The theoretical cross sections were computed using values listed in the NIST database. Optical cross sections were taken from UV-Vis spectra shown in Figure 1 b.

1. **Multiplet calculations**

To understand the possible origin of the transient Kα_1_ lineshapes multiplet calculations were performed in CTM4XAS 5.5 code^16^. This code allows in user-friendly way to analyze multiplet effects in various X-ray spectroscopies. In the current experiment focus was put on the 1s2p transitions (Kα XES), and atomic Hamiltonian parameters were not varied due to the limited valence sensitivity of core-to-core XES. Both [Fe-BL] and [Co] complexes separately and in the dyad are in distorted *O_h_* geometry, thus only 10Dq ligand field parameter, describing t_2g_ to e_g_ d-orbital splitting in octahedral field is critical. The calculations were divided into two steps:

1) determination of the 10Dq value required for the HS → LS transition (Fig. S3.6);

2) calculations of the ΔXES for combinations of possible electronic configurations;

3) optimization of the Slater integral overlap factors and broadening parameters (Fig. S3.7);

**Figure S3.6.** HS → LS transition 10Dq value: **a)** Fe Kα line evolution with increasing 10Dq value; **b)** Co Kα line evolution with increasing 10Dq value ; **c)** Intensity of the Fe Kα_1_ feature in function of 10Dq along with the step function fit (orange); **d)** Intensity of the Co Kα_1_ feature in function of 10Dq along with the step function fit (orange).

**a)**

**b)**

**c)**

**d)**

The initial 10Dq values for [Fe-BL] and [Co] were taken from ground-state TD-DFT calculations and were 5.0 eV and 4.6 eV, respectively. The values were calculated as difference between averaged energies of $d_{x^{2}-y^{2}}$, $d_{z^{2}}$, and $d_{xy}$, $d_{xz}$, $d_{yz}$ orbitals. However, TD-DFT tends to overestimate 10Dq value, as it does not take into account ligand-relaxing effects, that are empirically included in the crystal field multiplet theory.

The transition values of 10Dq for Fe^2+^ and Co^3+^ obtained from multiplet calculations were 2.20 eV and 2.49 eV, correspondingly. Since the real crystal field splitting values were unknown in final calculations the 10Dq values from TD-DFT were taken. During the optimization the Stalter integrals F_dd_, F_pd_ and G_pd_ were reduced to 0.66 for Fe and 0.5 for Co. For plotting the lorenzian broadening of 1.2 eV and 1.3 eV were taken for Fe and Co respectively, and gaussian broadening of 0.2 eV for both. The electronic configurations in Fig. S3.7 a-b were assigned based on the corresponding LF values. For iron moiety, the assignment is well known: upon excitation one electron is promoted to ligands, forming globally ^3^MLCT state (blue) with one unpaired electron on Fe atom. On the other hand, the ^3^MC is formed when two electrons are unpaired, either due to the SCO or due to back-CT from a ligand (red). For Co moiety, the situation is more complicated, namely there are two lineshapes: almost symmetric (dark blue) around t_0_ and strongly asymmetric (dark red) for longer delay times. Such shapes were identified for example in Ru^III^-Co^II^ dyads^17^ and for the quartet HS state of [Co(terpy)_2_]^3+^ ^18^. Thus, with our multiplet calculations we can assign the symmetric shape to the quartet Co^II^_._ The only possibility to reach this state is stepwise charge transfer to doublet Co^II^ followed by reduction of the ligand field splitting and SCO to quartet Co^II^ which occurred in the time frame of our temporal resolution (~120 fs). The second component marked as SCO in Fig. S3.7 was reproduced from spectral difference Co^3+^ _HS_ ­– Co^3+^_LS._ As such, this would be a metal centered state of unknown multiplicity. The relation between possible ^3^MC and ^5^MC states depends on the axial ligand situation. Detailed solution of this problem exceeds the scope of the current paper.

**Figure S3.7. a)** Fe ΔKα line lineshape calculation assuming MLCT (Fe^2+^_LS_ → Fe^3+^ _LS_, blue) and MC (Fe^2+^_LS_ → Fe^2+^ _HS_, red) states compared to the experimental data. Bottom: electronic spin configurations on the Fe ed shell assuming perfect octahedral geometry; **b)** Co ΔKα line lineshape calculation assuming CT (Co^3+^_LS_ → Co^2+^ _HS_, blue) in early dynamics (around t­_0_), and SCO state for later dynamics (Co^3+^_LS_ → Co^3+^ _HS_, red) compared to the experimental data at 50 fs and 3 ps, respectively. Bottom: electronic spin configurations on the Co d shell assuming perfect octahedral geometry. A possible CT (Co^3+^_LS_ → Co^2+^ _LS_, black) was also taken under account, however the obtained lineshape contradicts the experimental data. However, this lineshape corresponds to the transition state in CT1.

**a)**

**b)**

1. **Nuclear wavepacket analysis**

The oscillatory signals, extracted from the kinetic traces of both [Fe-BL] and [Fe-BL-Co] shown in Figure 4 of the main text, were analyzed in detail using the Fourier transform analysis and are presented in Fig S3.8a-b.

**a**

**b**

**c**

**Figure S3.8.** Fourier transform of the oscillatory parts: **a)** [Fe-BL]; **b)** Fe part of [Fe-BL-Co]. Raman spectra of [Co], [Fe-BL], and [Fe-BL-Co] obtained with 785 nm laser (**c)**).

For [Fe-BL], we found one dominating frequency of 3.49 THz (the corresponding half-period of 0.29 ps) and for the [Fe-BL-Co], there are two dominating frequencies of 3.71 THz (0.27 ps) and 5.56 THz (0.19 ps). These frequencies correspond to vibrational modes of 116.4 cm^-1^ (for [Fe‑BL]), 123.8 cm^-1^ and 185.5 cm^-1^ (for [Fe-BL-Co]), respectively, in the range of one degree of freedom for single bond thermal oscillations at 25 °C (*k_b_T*). Noteworthy, similar frequencies could be resolved experimentally by means of Raman spectroscopy (see spectra in Figure S3.8c). Specifically, the difference between 116.4 cm^-1^ and 123.8 cm^-1^ (~1 meV) could be observed only in terms of the amplitude, while a band around 185.5 cm^-1^ should appear only in [Fe-BL-Co]. It must be underlined, that in TR-XES such differences would be difficult to be detected. Since the phase of Fourier transform can be affected by the presence of a significant noise contribution, we decided to use an additional method of analysis. The nuclear wavepacket motion parameters for [Fe-BL-Co] were refined with the use of the damped oscillatory, function:

$f\left( t \right)=y_{0}+e^{\frac{-t}{t_{0}}}\left( b_{1}sin\left( \pi\frac{t-t_{c1}}{w_{1}} \right)+b_{2}sin\left( \pi\frac{t-t_{c2}}{w_{2}} \right) \right)$ (S3.c.1)

where:

*y_0_* – intensity offset in a.u.; *t_c1_, t_c2_* – time shifts in ps (which corresponds to phase shift);
*w_1_, w_2_* – oscillation half-period in ps;

*t_0_* – damping factor in ps; *b_1_, b_2_* – initial amplitude of oscillations in a.u.

The results are presented below. In the case of [Fe-BL], the b_2_ was fixed to 0. It is worth mentioning that for [Fe-BL-Co] the damping factor is much bigger, although the uncertainty of this parameter prevents from deriving any quantitative conclusions. Interestingly, the oscillation in [Fe-BL] changes phase upon cobalt coordination. This may suggest, that after forming the dyad, the longer oscillation is partially quenched and re-induced upon photoexcitation. The phase of oscillation in [Fe-BL] is ~ 9° (=t_c1_w_1_^-1^×180^o^π^−1^), i.e. close to sinusoidal, therefore induced by impulse-stimulated Raman scattering.^19–21^ On the other hand, the 0.255 ps component phase in [Fe-BL-Co] is ~51°, thus of mixed sine/cosine character and for 0.185 ps, the phase with ~89° is of cosine character. The cosine type of oscillation was proven to be a direct marker of the influence of the excited state generation upon photoexcitation and followed by coherent vibrations generation ^21,22^. Thus at least one of the vibrations in [Fe-BL-Co] is related to the charge transfer accompanied by the Fe-ligand bond stretching.

**Table S3.2.** Wavepacket analysis fitting results with equation (S3.c.1).

| **[Fe-BL]** | | **[Fe-BL-Co]** | |
| --- | --- | --- | --- |
| b_1_ | 0.064(176) | b_1_ | -0.008(4) |
| tc_1_ [ps] | 0.046(17) | tc_1_ [ps] | 0.082(9) |
| w_1_ [ps] | 0.284(22) | w_1_ [ps] | 0.255(30) |
| $y_{0}$ | 0.003(4) | b_2_ | 0.015(4) |
| t_0_ [ps] | 0.402(150) | tc_2_ [ps] | 0.100(4) |
|  |  | w_2_ [ps] | 0.185(8) |
|  |  | $y_{0}$ | 0.000(2) |
|  |  | t_0_ [ps] | 1.632(1.270) |
|  |  |  |  |

**Figure S3.9. a)** Single oscillation model fitting to wavepacket signal; **b)** F distribution (black) for nested 5-parameter model in 8-parameter model along with F-value in this study (blue). The vertical line indicates critical F-value of 3.6875 above which the null hypothesis can be rejected in the current conditions at p level of 0.05.

**a**

**b**

Given the large experimental error bars, we wanted to discard the possibility that the observed additional oscillation for the dyad could compensate for the high damping value. In other words, we have also verified the scenario, in which the extracted oscillations could be described with a single sine function. These fit results are shown in Figure S3.9a, and compared statistically with the double sine fit, previously presented in Figure 4a, using an F-test (Figure S3.9b). The F-test is intended to compare two models, where one of them contains less parameters and is nested into a more complex one. The result of the test indicates statistical significance of the more complex fit, especially when χ^2^ comparison is not enough, thus preventing us from overfitting the data. In our specific case, the single-sine model was described by 5 parameters ([Fe-BL] column in Table S3.2) and was nested into a more complex model described with 8 parameters ([Fe-BL-Co] column in Table S3.2). The fitting procedure was carried out with experimental error bars as weights, and in F-value calculation, χ^2^ values of 0.01338 and 0.00814 were found for single- and double-sine fit functions, respectively. Since all fits were done with p = 0.05, the null hypothesis in this test stated that fitted functions are not different in 95% probability. For such conditions, the critical F-value to reject null at 95% probability was 3.6875, while the obtained value was 5.0053. Therefore, we can conclude that both models are statistically different and thus confirm the presence of the second oscillation in [Fe-BL-Co] kinetic traces.

1. **Co Kα_1_ kinetic signals for -5-15 ps time window**

The statistics of the Co Kα_1_ long-time kinetic signal in [Fe-BL-Co] is substantially worse than for pure cobaloxime measurement, owing mainly to the two factors. First of all, due to substantially different absorption cross sections in the UV-Vis range, upon the photoexcitation of the dyad, the [Fe-BL] part is predominantly excited, while the direct excitation of the Co part is nearly completely avoided. Secondly, the X-ray beam intensity is distributed over two metal centers. Since with the measurement of cobaloxime alone, the aim was to detect excited states formed upon direct photoexcitation, and no [Fe-BL] was present. Still, the long kinetic trace for [Fe-BL-Co] substantially differs in shape as compared to pure cobaloxime, especially in the initial 5-6 ps range (sec. 3a, Figure S3.5 a, and c). Consequently, although a possible long-lived component in Co moiety could not be excluded, it is visible only after the first ~7 ps of the [Fe-BL-Co] Co Kα_1_ kinetics, where the signal almost reaches the background level. It would not affect the presence of *τ_1,Fe_****_Co_*_,_** and short-time kinetic trace, since contributions from the shortest time constants appear at the beginning of the kinetic evolution. Moreover, the step size in this measurement was equal to 1 ps, which corresponds to approx. 2 data points that represent time constant of 1 ps. The initial fitting results of the kinetic traces for Co Kα fluorescence in [Fe-BL-Co] in 15 ps time window are shown in Figure S3.5 a. All fit parameters were left as free and as a result we obtained good fit results with two time constants of 1.4 and 17 ps, respectively. The result is shown in Figure S3.10 and the corresponding parameters are summarized in Table S3.3 (column A). Interestingly, the fitting of Co Kα fluorescence to the 1.2 ps kinetic trace revealed unambiguous presence of another ultrashort contribution of 0.25 ps. The 1 ps time constant in the 15 ps kinetic fit, was statistically represented by a single point, therefore it could be an artefact. To verify this, we repeated the fitting of the Co Kα fluorescence to the 15 ps kinetic trace using a fixed time constant of 0.25 ps and keeping all other fit conditions the same. However, the fit did not converge to any reasonable result, and therefore we extended the fit model with an additional time constant. First, we fitted all 3 decays, and the fitting procedure produced a very large time constant and high uncertainties. This contribution was interpreted as a representation of an electronic state with decay significantly longer than the time window of measurement. Therefore, the large value was fixed, and data were re-fitted. The result is shown in Figure S3.10 b and corresponding parameters are available in Table S3.3 under column B. The results again exhibit 1 ps time constant (and did not require the shortest 0.25 ps time constant), which was interpreted as an artefact and a time constant of 7 ps with a very high uncertainty. For the purpose of testing this hypothesis further, we employed a fit procedure with three time constants, of which two: 0.25 ps and infinite were fixed. The result is shown in Figure S3.10 c and corresponding parameters are available in Table S3.3 under column C. The results concluded that the 1 ps value from the first fitting attempt was indeed an artefact due to too low temporal step size of the measurement. Earlier reports suggest that a two-exponential, sequential decay for cobaloxime (one fast and second around 20 ps), and we assumed the same scenario for our longer time constants.^23^ Therefore, *A_2_*, *τ_2_*, *A_3_*, and *τ_3_* represented a decay of the LMCT/SCO state to the ground state through the MC state upon direct photoexcitation, while *τ_1_* and *A_1_* represented M’MCT transition. This implied that the corresponding amplitudes of the *A_2_* and *A_3_* in the model from Table S3.3 C will be similar because both concern excited states in the same simple decay pathway. However, the difference between them is around a factor of 2. Due to the fact that the LMCT excitation is still present, we assumed that the related decay pathway will correspond to the one in an isolated cobaloxime complex, especially for the lowest-lying state. A final fitting attempt was conducted on the model described in Table S3.3 C, with *τ_3_* fixed at the value obtained from cobaloxime, namely 23.39 ps. The result is presented in Figure S3.5A and Table S3.1. Notably, the relation between *A_2_* and *A_3_* is almost 1. The time constant of 4.1 ps is discussed in the main text. The value of *τ_3_* was later re-evaluated with other time constants fixed, and a value of 29.39(14.46) ps was obtained.

**Table S3.3.** Co Kα fluorescence decay fitting results for [Fe-BL-Co] with different assumptions.

| **A** | | **B** | | **C** | |
| --- | --- | --- | --- | --- | --- |
| A_1_ | 4.453(1.535) ·10^-4^ | A_1_ | 4.101(844) ·10^-4^ | A_1_ | 3.160(116) ·10^-3^ |
| τ_1_ [ps] | 1.36(25) | τ_1_ [ps] | 1.16(50) | τ_1_ [ps] | 0.25 (fixed) |
| A_2_ | 1.914(103) ·10^-4^ | A_2_ | 1.874(757) ·10^-4^ | A_2_ | 1.967(114) ·10^-4^ |
| τ _2_ [ps] | 16.74(3.54) | τ _2_ [ps] | 7.11(5.67) | τ _2_ [ps] | 5.97(1.19) |
| t_0_ [ps] | -0.56(7) | A_3_ | 5.993(4.267) ·10^-5^ | A_3_ | 6.393 (117) ·10^-5^ |
| $y_{0}$ | 1.041(48) ·10^-5^ | τ _3_ [ps] | infinite (fixed) | τ _3_ [ps] | infinite (fixed) |
| FWHM^a^ [ps] | 0.284 (fixed) | t_0_ [ps] | -0.57(5) | t_0_ [ps] | -0.01(2) |
|  |  | $y_{0}$ | 1.025(25) ·10^-4^ | $y_{0}$ | 1.024(25) ·10^-4^ |
|  |  | FWHM^a^ [ps] | 0.284 (fixed) | FWHM^a^ [ps] | 0.284 (fixed) |

**b)**

**c)**

**Figure S3.10.** Co Kα fluorescence decay fitting results for [Fe-BL-Co] 15 ps kinetics: a) with 2 time constants; b) with 3 time constants and very long time constant fixed; c) with 3 time constants and 0.25 ps and infinite time constants fixed.

**a)**

1. ***d*-*d* interactions in Co**

The *d*-*d* interactions can occur when different valence *d* orbitals in a metal complex are not fully occupied. In octahedral symmetry, the selection rules forbid *d*-*d* transitions to occur and thus they can be observed when the symmetry of the complex is significantly distorted, for example by the Jahn-Teller effect. Even though the symmetry of Co(dmgH)_2_Cl(py) complex is significantly distorted, thus allowing weak *d*-*d* transitions to occur, the UV-Vis spectrum of pure cobaloxime does not contain the characteristic *d*-*d* bands (Figure 1 b) observed for another family of cobaloximes with axial alkyl and amino ligands [Co(dmgH)_2_(Alkyl)(Base)]^24^. Therefore, the low-lying acceptor state for LMCT deexcitation is not populated due to the *d*-*d* transition according to the UV-VIS. In order to assign the time constants obtained via the fitting of TR-XES kinetic traces to possible transitions in our Co complex, the *τ_1,Co_* was tentatively assigned as LMCT → MC decay, while *τ_2,Co_* represent the MC relaxation in this model. We want to underline, that the MC nature of the second state must be independently confirmed, yet the LMCT state was also confirmed by DFT results, making MC state an obvious candidate as an acceptor for the LMCT decay. On the other hand, the multiplet calculations suggest SCO state. Thus, we do not exclude it and the proposed state diagram for the relaxation on the Co site of the dyad is also analogical to numerous Fe polypyridyl complexes with long-lived MC states.^25,26^ Additionally we assign the long-lived state as LMCT/SCO. A dedicated Co Kβ TR-XES experiment would further confirm the nature of the assigned states involved in the decay process, which was beyond the scope of the present study.

1. **Kinetic model for XES fluorescence kinetic traces.**

*Fe Kα XES.* For Fe in [Fe-BL-Co], two decay channels were proposed (Figure 5):

1) alpha (α) channel: ^1/3^MLCT* $\underset{\to}{k_{1}}$ ^3^MLCT $\underset{\to}{k_{2}}$ ^3^MC $\underset{\to}{k_{3}}$ gs;

2) beta (β) channel: ^1/3^MLCT* $\underset{\to}{k_{4}}$ ^3^MC $\underset{\to}{k_{3}}$ gs.

They were described by a system of differential equations:

$\frac{d{}^{1}{MLCT}}{dt}=-k_{1}{}^{1}{MLCT}-k_{4}{}^{1}{MLCT}$ (S3.1.a)

$\frac{d{}^{3}{MLCT}}{dt}=k_{1}{}^{1}{MLCT}-k_{2}{}^{3}{MLCT}-k_{ct}{}^{3}{MLCT}$ (S3.1.b)

$\frac{d{}^{3}{MC}}{dt}=k_{4}{}^{1}{MLCT}+k_{2}{}^{3}{MLCT}-k_{3}{}^{3}{MC}$ (S3.1.c)

$gs=M-{}^{1}{MLCT}-{}^{3}{MLCT}-{}^{3}{MC}$ (S3.1.d)

${}^{1}{MLCT(t=0)}=M$ (S3.1.e)

${}^{3}{MLCT(t=0)}=0$ (S3.1.f)

${}^{3}{MC(t=0)}=0$ (S3.1.g)

where: *M* – initial excited state fraction. For the modelling purposes, an equal relation between concentration and signal strength was assumed. The system of differential equations above was solved numerically in Mathematica 11 software and all solutions were broadened by Heaviside step function under the convoluted with normalized Gaussian function to model rise time of electronic state:

$g_{broadened}\left( t \right)=\frac{1}{2\sigma\sqrt{2\pi}}\int e^{-\frac{y^{2}}{2\sigma^{2}}}h(t-t_{0}-y)g(t)dy$ (S3.3)

where:

$g(t)$ – broadened function defined by one of eq. S3.1.a – S3.1.g;

$h(t-t_{0}-y)$ – Heaviside step function;

*σ* – Gaussian standard deviation function.

The final fitted function was as follows:

$f_{Fe}\left( t \right)=y_{0}+{}^{1}{MLCT}+{}^{3}{MLCT}+{}^{3}{MC}$ (S3.4)

where:

$y_{0}$ – vertical offset.

*Co Kα XES.* There were two decay paths identified in [Co]:

1) M’MCT $\underset{\to}{k_{7}}$ gs;

2) LMCT $\underset{\to}{k_{5}}$ MC $\underset{\to}{k_{6}}$ gs.

The charge transfer (CT) was treated as instantaneous, therefore it is completed within the IRF function. The M’MCT state was acting as an acceptor of CT from bridging ligand BL, while LMCT/SCO (designated as LMCT in the equations below) state was representing direct optical excitation and was described by an independent *k_5_* rate constant. The analogical transition was observed in pure cobaloxime kinetic data with 2.76 ps time constant. The differential formula system with boundary conditions was as follows:

$\frac{dM'MCT}{dt}=-k_{7}M'MCT$ (S3.5.a)

$\frac{dLMCT}{dt}=-k_{5}LMCT$ (S3.5.b)

$\frac{dMC}{dt}=k_{5}LMCT$ - $k_{6}MC$ (S3.5.c)

${gs}_{Co}={M'MCT}_{0}+{LMCT}_{0}-M'MCT-LMCT$ (S3.5.d)

$M'MCT\left( t=0 \right)={M'MCT}_{0}$ (S3.5.e)

$LMCT\left( t=0 \right)={LMCT}_{0}$ (S3.5.f)

The final fitting function was:

$f_{Co}\left( t \right)=y_{0}+M'MCT+LMCT+MC$ (S3.6)

The $M'MCT$ and $LMCT$ were also broadened by the function described in eq. 3.

All fitting results are summarized in Table S3.4. In the first approach, decay constant values obtained from the fluorescence decay formula fitting were fixed. The amplitudes and offsets were fitted. Afterward obtained values were fixed to refine the rate constants.

**Table S3.4.** Summary for fitting kinetic equations to experimental data.

| **Fe in [Fe-BL] // Fe in [Fe-BL-Co]** | | | **[Co] in [Fe-BL-Co] dyad // cobaloxime** | | |
| --- | --- | --- | --- | --- | --- |
| $M$ | 0.736(19) | 0.608(9) | ${M'MCT}^{0}$ | 0.966(160) | - |
|  |  |  | ${LMCT}^{0}$ | 0.730(64) | 0.925(68) |
| t_0_ [ps] | -0.034(8) | 0.002(0.0005) | t_0_ [ps] | -0.005(0.015) | -0.018(0.021) |
| $y_{0}$ | 0.047(14) | 0.027(0.007) | $y_{0}$ | 0.002(0.032) | 0.004(0.048) |
| k_1_ / τ_1_ [ps^-1^ /ps] | 4.527(1.495) / 0.221(72) | 4.527 (fixed) / 0.221 (fixed) | k_5_ / τ_5_ [ps^-1^/ ps] | 4.12 (fixed) | 2.76 (fixed) |
|  |  |  |  |  |  |
| $k_{et}$/ τ_et_ [ps^-1^ /ps] | - | -0.294(35) / -3.404(405) | k_6_ / τ_6_ [ps^-1^ /ps] | 3.659(1.246) / 0.273(93) | - |

The *k_2_*, *k_3_*, *k_4_*, and *k_5_* rate constants were calculated from *k = τ^-1^* relation. Decay time constants *τ* were taken from fluorescence fitting results. In total, five parameters were fitted to the experimental data: *M*, *y_0_*, *t_0_*, *k_1_* and *k_et_*. Unlike for the FWHM value used in the fluorescence fitting, the *σ* parameter here is a standard deviation of Gaussian in the IRF function. The re-evaluated value of *σ* = 0.106(5) ps corresponds to 0.25(1) ps from the fluorescence fitting, which clearly resembles the FWHM of IRF function (0.28 ps). The *t_0_* was set as a free parameter to ensure fit convergence. The *k_1_* decay rate represents a ^1^MLCT->^3^MLCT transition, and the inclusion of this decay rate is necessary due to nuclear wavepacket motion shown in Figure 4 and Figure S3.5. According to DFT results, eventual charge transfer should go through BL ligand acceptor state. The CT in the [Fe-BL] part of the dyad was considered to originate from two possible states: the ^3^*MLCT* or ${}^{3}{{MLCT}}$. In both cases, to model CT, an additional element of *–k_ct_*${}^{3}{{MLCT}^{*}}$ or *–k_ct_MLCT* was added into differential equations S3.1.b and S3.1.a respectively. In the first case, any fitting attempts were unsuccessful. For CT from ^3^MLCT state, a two-step analysis was applied. First, for [Fe-BL] a rate constant *k_1_* for ^1^MLCT$\to$ ^3^MLCT transition was evaluated. For CT from ^3^MLCT in [Fe-BL-Co] this value was fixed, and the fit was conducted with the free *k_ct_* parameter. Results for global kinetic fitting are presented in Figs. 5, S3.11 and S3.12.

In the case of cobaloxime analysis, a two-state decay model was used to match the fluorescence decay results. Two scenarios were included, where states either decay in parallel or in a hierarchical way. Only the hierarchical model was reproducing the data with the states diagram (Figure S3.13) and fitted kinetic traces presented in Figure S3.10. In early kinetics (-1-1 ps), the signal dynamics can be described by 2.76 decay time, while a longer period requires the inclusion of a second, infinite time constant. This model was the basis for [Fe-BL-Co] analysis with 4.12 ps time fixed in a short-time window (Figure S3.5). Importantly, the dynamics in short- and long-time windows could be reproduced with two kinetic constants. The results of kinetic equation fitting (Fig 5 c, Figure S3.12 b) confirmed the fluorescence kinetic trace analysis for Co in [Fe-BL-Co]. For the sake of precision, two models were also tested: the M’MCT and LMCT states decaying in a hierarchical and parallel way. Only the model presented in Figure 5 reproduced data with satisfactory quality. Due to a very low Co signal intensity in [Fe-BL-Co] the estimated uncertainties are high.

**Figure S3.12.** Populations for short- and long-time window kinetic traces for cobaloxime: **a)** long-time window; **b)** short-time window.


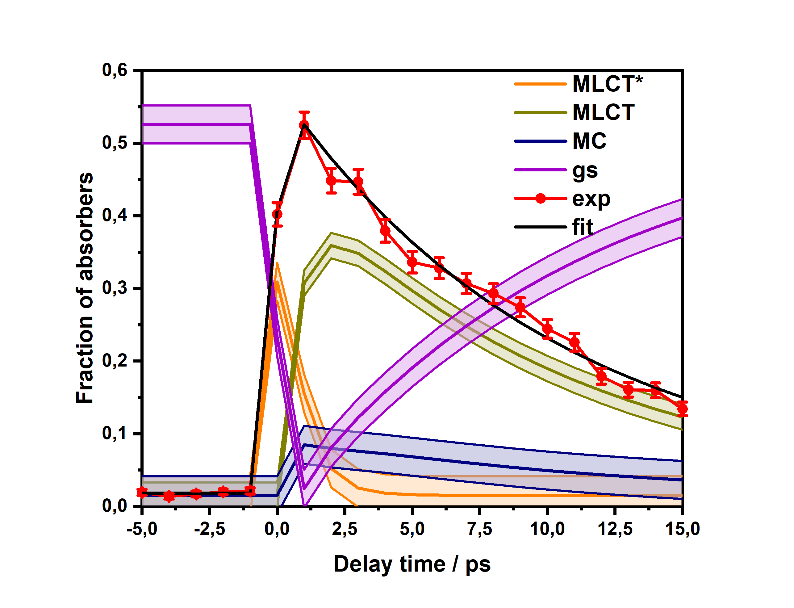


**Figure S3.11.** Total kinetic traces for: **a)** [Fe-BL], short-time window; **b)** Co @ [Fe-BL-Co], long-time window; **c)** [Fe-BL], long-time window; **d)** Fe @ [Fe-BL-Co], long-time window.

**a)**

**b)**

**c)**

**d)**

**Figure S3.13.** State diagram for: **a)** [Fe-BL] and **b)** cobaloxime.

**a)**

**b)**


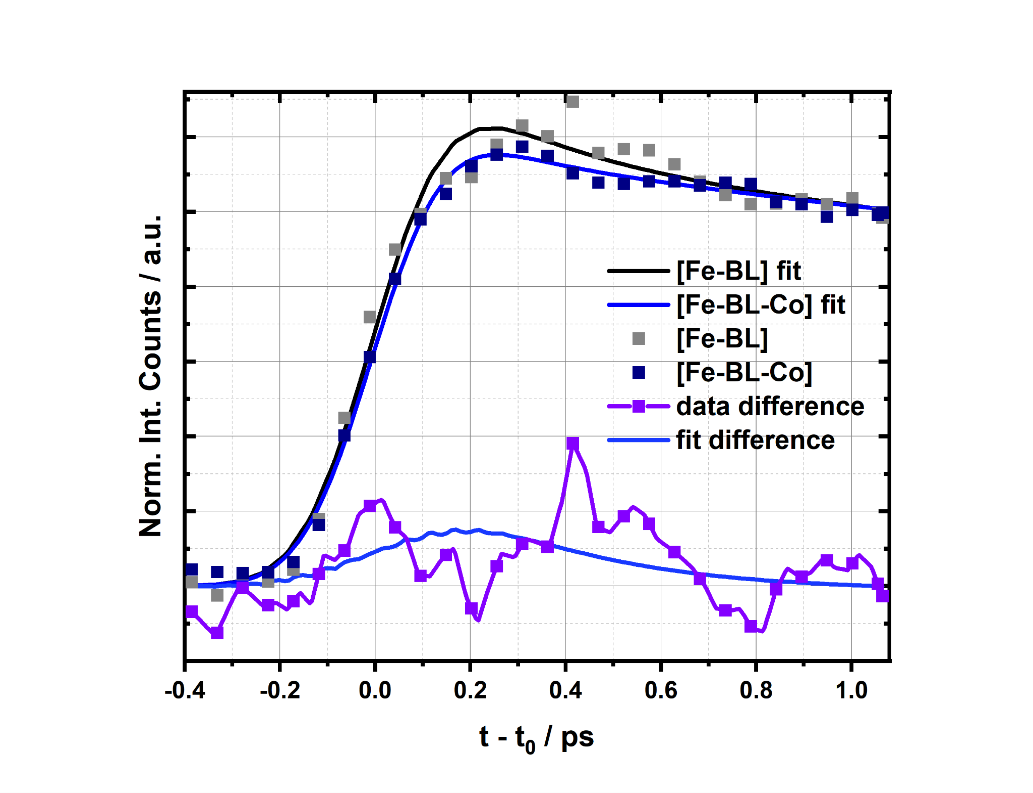


**Figure S3.14.** Comparison of the kinetic traces and corresponding fits for Fe in [Fe-BL] (grey and black) and [Fe-BL-Co] (light blue and navy). Small changes between the kinetic traces are more difficult to track in respect to the Co data.

**References:**

1. Dreuw, A. & Head-Gordon, M. Failure of Time-Dependent Density Functional Theory for Long-Range Charge-Transfer Excited States: The Zincbacteriochlorin-Bacteriochlorin and Bacteriochlorophyll-Spheroidene Complexes. *J Am Chem Soc* **126**, 4007–4016 (2004).

2. Autschbach, J. Charge-transfer excitations and time-dependent density functional theory: Problems and some proposed solutions. *ChemPhysChem* **10**, 1757–1760 (2009).

3. Vlček, A. & Záliš, S. Modeling of charge-transfer transitions and excited states in d6 transition metal complexes by DFT techniques. *Coord Chem Rev* **251**, 258–287 (2007).

4. Khedkar, A. & Roemelt, M. Modern multireference methods and their application in transition metal chemistry. *Physical Chemistry Chemical Physics* **23**, 17097–17112 (2021).

5. González, L., Escudero, D. & Serrano-Andrés, L. Progress and challenges in the calculation of electronic excited states. *ChemPhysChem* **13**, 28–51 (2012).

6. Dreuw, A. & Head-Gordon, M. Single-reference ab initio methods for the calculation of excited states of large molecules. *Chem Rev* **105**, 4009–4037 (2005).

7. Liu, Z., Lu, T. & Chen, Q. An sp-hybridized all-carboatomic ring, cyclo[18]carbon: Electronic structure, electronic spectrum, and optical nonlinearity. *Carbon N Y* **165**, 461–467 (2020).

8. Lu, T. & Chen, F. Multiwfn: A multifunctional wavefunction analyzer. *J Comput Chem* **33**, 580–592 (2012).

9. Hainer, F. *et al.* Vibrational Coherence Spectroscopy Identifies Ultrafast Branching in an Iron(II) Sensitizer. *J. Phys. Chem. Lett.* **12**, 8560–8565 (2021).

10. Tatsuno, H. *et al.* Hot Branching Dynamics in a Light-Harvesting Iron Carbene Complex Revealed by Ultrafast X-ray Emission Spectroscopy. *Angewandte Chemie - International Edition* **59**, 364–372 (2020).

11. Kunnus, K. *et al.* Vibrational wavepacket dynamics in Fe carbene photosensitizer determined with femtosecond X-ray emission and scattering. *Nat Commun* **11**, 1–11 (2020).

12. Canton, S. E. *et al.* Visualizing the non-equilibrium dynamics of photoinduced intramolecular electron transfer with femtosecond X-ray pulses. *Nat Commun* **6**, 1–10 (2015).

13. Pápai, M., Vankó, G., Rozgonyi, T. & Penfold, T. J. High-Efficiency Iron Photosensitizer Explained with Quantum Wavepacket Dynamics. *Journal of Physical Chemistry Letters* **7**, 2009–2014 (2016).

14. Fredin, L. A. *et al.* Exceptional excited-state lifetime of an iron(II)- N -heterocyclic carbene complex explained. *Journal of Physical Chemistry Letters* **5**, 2066–2071 (2014).

15. Léonard, J. *et al.* High-throughput time-correlated single photon counting. *Lab on a Chip - Miniaturisation for Chemistry and Biology* **14**, 4338–4343 (2014).

16. Stavitski, E. & de Groot, F. M. F. The CTM4XAS program for EELS and XAS spectral shape analysis of transition metal L edges. *Micron* **41**, 687–694 (2010).

17. Canton, S. E. *et al.* Visualizing the non-equilibrium dynamics of photoinduced intramolecular electron transfer with femtosecond X-ray pulses. *Nature Communications 2015 6:1* **6**, 1–10 (2015).

18. Canton, S. E. *et al.* Ultrafast Jahn-Teller Photoswitching in Cobalt Single-Ion Magnets. *Advanced Science* **10**, (2023).

19. Yan, Y. X. & Nelson, K. A. Impulsive stimulated light scattering. I. General theory. *J Chem Phys* **87**, 6240–6256 (1987).

20. Yan, Y. X., Gamble, E. B. & Nelson, K. A. Impulsive stimulated scattering: General importance in femtosecond laser pulse interactions with matter, and spectroscopic applications. *J Chem Phys* **83**, 5391–5399 (1985).

21. Ishioka, K., Kitajima, M. & Misochko, O. V. Coherent A1g and Eg phonons of antimony. *J Appl Phys* **103**, (2008).

22. Naumova, M. *et al.* Revealing Hot and Long-Lived Metastable Spin-States in the Photoinduced Switching of Solvated Metallogrid Complexes with Femtosecond Optical and X-ray Spectroscopies. *J Phys Chem Lett* acs.jpclett.9b03883 (2020) doi:10.1021/acs.jpclett.9b03883.

23. Mulfort, K. L. Interrogation of cobaloxime-based supramolecular photocatalyst architectures. *Comptes Rendus Chimie* **20**, 221–229 (2017).

24. Ikeda, K. *et al.* Photo-induced chirality switching in a cobaloxime complex crystal. *Journal of Chemical Physics* **122**, 1–5 (2005).

25. Monat, J. E. & McCusker, J. K. Femtosecond excited-state dynamics of an iron(II) polypyridyl solar cell sensitizer model. *J Am Chem Soc* **122**, 4092–4097 (2000).

26. Kunnus, K. *et al.* Tuning excited state electron transfer in Fe tetracyano-polypyridyl complexes. *ArXiv* 1–21 (2019).
